# Neonatal reference intervals for salivary steroids: focus on prematurity, stressful events and perinatal betamethasone

**DOI:** 10.3389/fped.2026.1808446

**Published:** 2026-06-16

**Authors:** Svetlana N. Zykova, Anna Plaksienko, Anne Lee Solevåg, Anne Karin Brigtsen, Trond Melbye Michelsen, Ingvil Krarup Sørbye, Mariann Haavik Lysfjord Bentsen, Liv Hanne Bakke, Julie Enge Elboth, Jens Petter Berg, Sandra Rinne Dahl, Per Medbøe Thorsby

**Affiliations:** 1 Hormone Laboratory, Department of Medical Biochemistry, Endocrinology and Metabolism Research Group, Oslo University Hospital, Oslo, Norway; 2 The Public Dental Health Service Competence Centre of Northern Norway, Tromsø, Norway; 3 Oslo Centre of Biostatistics and Epidemiology, University of Oslo, Oslo, Norway; 4 Department of Neonatal Intensive Care, Oslo University Hospital, Oslo, Norway; 5 Institute of Clinical Medicine, University of Oslo, Oslo, Norway; 6 Department of Obstetrics, Oslo University Hospital, Oslo, Norway; 7 Department of Paediatrics, Haukeland University Hospital, Bergen, Norway; 8 Department of Medical Biochemistry, Oslo University Hospital, Oslo, Norway

**Keywords:** adrenal steroid hormones, neonatal intensive care unit, preterm/full term infants, reference intervals (RIs), saliva, betamethasone, antenatal corticosteroids, 17OH-pregnenolone/17OH-progesterone/11-deoxycortisol/21-deoxycortisol/cortisone/deoxycorticosterone/corticosterone/aldosterone/DHEA/DHEAS/androstenedione/testosterone

## Abstract

**Context:**

Painlessly obtained samples can improve diagnostics and care in paediatric conditions with affected steroidogenesis.

**Objective:**

To establish neonatal reference intervals (RIs) and identify sources of biological variation for steroid hormones in saliva.

**Methods:**

Steroid concentrations were determined using LC-MS/MS in 412 saliva samples from 174 infants aged 0-4 weeks, born at gestational age 26–42 weeks, recruited from three neonatal intensive care units and one maternity ward. We used six approaches to construct RIs with partitioning based on results from linear mixed models.

**Results:**

RIs in nmol/L calculated with robust method using median of up to six samples with sufficient volume from each infant were for salivary 17OH-pregnenolone: <4.0; 17OH-progesterone: <2.5; 11-deoxycortisol: 0.02–0.99; 21-deoxycortisol: <0.2; cortisol: 0.65–39; cortisone: 18–154; deoxycorticosterone: <0.04; corticosterone: <0.76; aldosterone: 0.21–4.2; dehydroepiandrosterone: <2.4; dehydroepiandrosterone sulphate: <100; androstenedione: <1.5; testosterone girls: <0.10; testosterone boys: <0.32. Following multivariable adjustment, prematurity was associated with elevated concentrations of mineralocorticoids, androgens and glucocorticoid-precursors (up to 240%, 500% and 1060% respectively), but not cortisol or cortisone. Being on respiratory support was linked with qualitative and quantitative changes in all corticosteroid pathways, while sampling during the first 48 h of life was associated with an increase in adrenocorticotropin-regulated glucocorticoids and androgens (except dehydroepiandrosterone) but not mineralocorticoids. Perinatal exposure to synthetic glucocorticoids led to 33% reduction in salivary cortisol, but not other hormones. Betamethasone was detectable in infants' saliva up to 12 days following maternal administration.

**Conclusions:**

We present neonatal reference intervals for steroid hormones in saliva thereby supporting a non-invasive and painless option for obtaining a steroid hormone profile in this fragile population.

## Introduction

**Figure 1:**
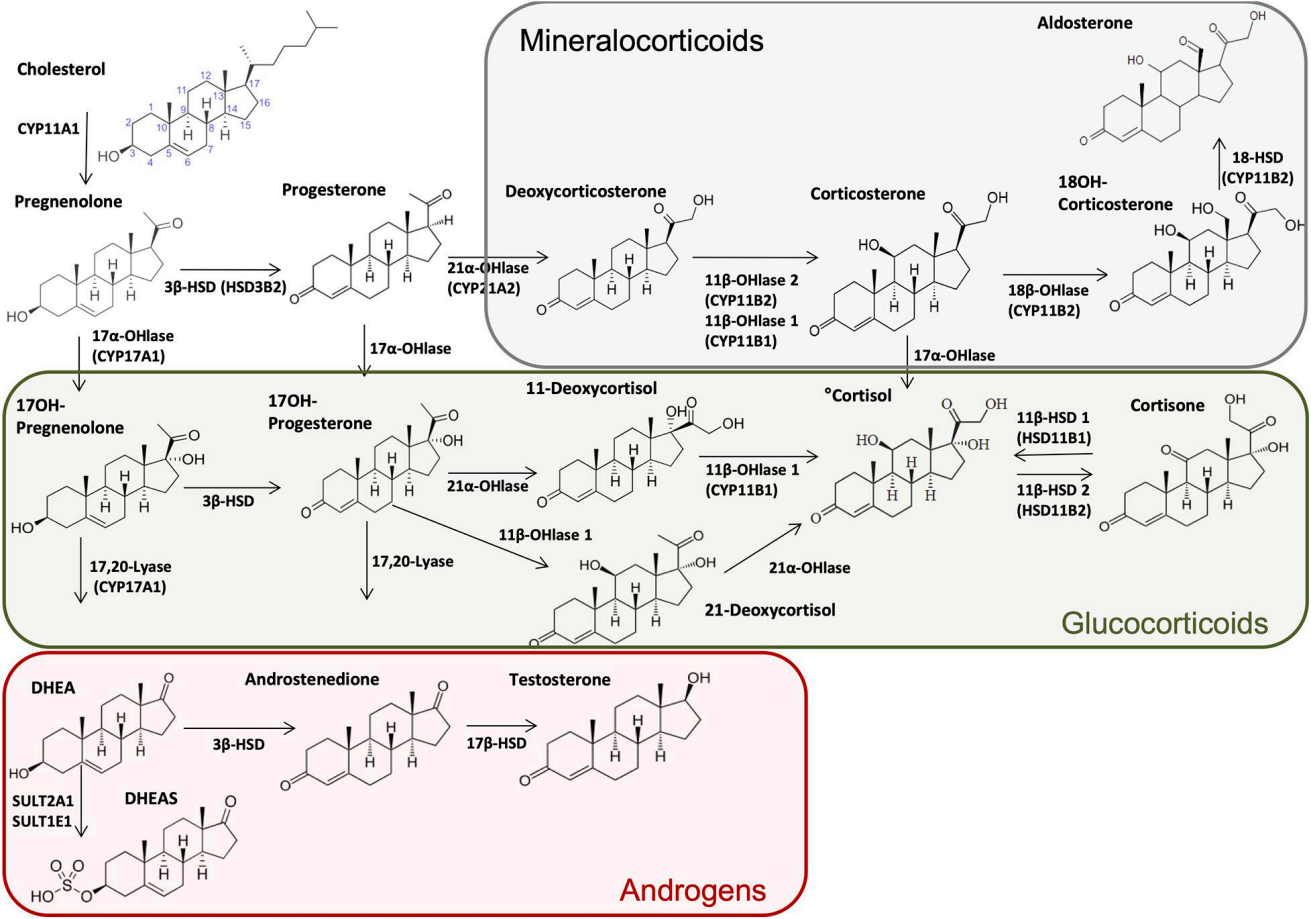
Adrenal steroidogenic pathways.

Diagnostic approaches to aberrations in steroidogenic pathways (Figure 1) often require simultaneous assessment of several hormones and their precursors. Relative overabundances of upstream to downstream metabolites point to enzymatic defects that manifest biochemically with characteristic signatures or patterns. Because production of some but not all adrenal hormones is increased in stress and anxiety, these conditions can change steroid profile both qualitatively and quantitatively. Sample matrices obtained by non-invasive painless procedures, such as saliva, therefore have apparent diagnostic and health-economic advantages. Importantly, saliva is a more attractive alternative to blood sampling from the user perspective, particularly in neonatology where the patients are subjected to repeated painful procedures and are often too small and vulnerable for frequent blood draws (1). Establishment of reference intervals and implementation of salivary adrenal panels in the clinical routine can improve patient care and close several knowledge gaps in this age group.

One of the most common inherited endocrine conditions - congenital adrenal hyperplasia (CAH) - is caused by impaired activity of one or several steroidogenic enzymes. The incidence of its severe classical form ranges from 1 in 282 in Yupik Innuits to 1 in 14000–18000 of live births worldwide (2, 3). The non-classical form gives milder phenotypes but is substantially more frequent with a prevalence of 1:500 to 1:1000 in the general population and up to 1:50 in groups with high rates of consanguineous marriages (4, 5). Early - and ideally pain-free - diagnostic approach to the classical CAH is needed as clinical manifestation in the form of life-threatening adrenal crisis is triggered by stress and occurs within the first three weeks of life in 75% of cases (6). Even though neonatal screening has improved early diagnostics of CAH, these patients require life-long treatment with synthetic steroids, that starts at infancy and needs careful monitoring (5, 7). Chronic over- and under-dosing of glucocorticoids in the treatment of CAH may increase morbidity and risk of anthropometric deviations, while physical stress can be lethal if the treatment is not adjusted concurrently (5, 7). Targeting replacement therapy to approximate natural diurnal patterns in adrenal steroids can improve clinical and biochemical outcomes in CAH, but the evidence so far is insufficient (8). There is no consensus on what laboratory markers to use as indicators of good control (9), but consistently timed serum 17OH-progesterone, androstenedione, testosterone and dehydroepiandrosterone sulphate (DHEAS) have been advocated (3). Several small studies, including one longitudinal, established the value of salivary cortisol and/or 17OH-progesterone in monitoring treatment in CAH, but there is still a need to establish age-specific reference intervals, to correct abnormal steroidogenesis beyond 17OH-progesterone and to tailor decision limits to patients' developmental needs (10–14).

Obtaining a complete and repetitive adrenocortical profile is also relevant in the context of preterm birth where the immaturity of enzymatic pathways and organogenesis makes differential diagnostics of CAH difficult (15). Furthermore, in high-income settings most infants born before 34 weeks' gestational age (GA) are exposed to synthetic steroids, either antenatally to accelerate lung maturation and/or postnatally to wean off respiratory support. Systemic treatment with glucocorticoids can cause tertiary adrenal insufficiency (16). Adults who are at risk of this condition are normally monitored with blood-based cortisol and adrenocorticotropin (ACTH) tests (17). Due to perils of phlebotomy and risk of iatrogenic anaemia, blood sampling in this pre-term group is not considered a hazard-free option (1), and screening for adrenal suppression is not routinely performed. Moreover, higher precursor-to-product ratios in the glucocorticoid pathway in prematurely compared to term-born neonates lowers the predictive value of cortisol in neonatal adrenal insufficiency (16) and other pathologies (18). Changes in adrenal hormones in response to procedural pain (19), even from minor procedures, such as blood sampling (20) adds to the complexity of the matter.

Saliva samples are obtained non-invasively and show superior stability for several adrenal steroids (21), making pre-analytical processing and shipment easier compared to that of blood. Saliva is also principally free of binding proteins (22), allowing determination of free and thus biologically active cortisol and testosterone. Among the disadvantages is insufficient standardisation of specimen collection procedures across institutions and potential effect of bleeding from oral mucosa on cortisol, dehydroepiandrosterone (DHEA) and testosterone concentrations (23).

Recently, a liquid chromatography–tandem mass spectrometry (LC-MS/MS) method for determination of 13 adrenal hormones and two synthetic steroids in low saliva volumes (2–50 µL) tailored specifically to neonatal needs has been developed and validated (21). Here we report reference intervals for salivary 17OH-pregnenolone, 17OH-progesterone, 11-deoxycortisol, 21-deoxycortisol, cortisol, cortisone, deoxycorticosterone, corticosterone, aldosterone, DHEA, DHEAS, androstenedione and testosterone for premature and term-born infants aged 0–4 weeks, and present exploratory analyses of anthropometric and clinical factors associated with salivary steroid concentrations. We also present data on betamethasone concentrations in saliva of newborn infants with prenatal exposure to the drug.

## Methods

### Design and settings

This diagnostic study with cross-sectional design and repeated saliva sampling (1-6 events) from infants admitted to Maternity Unit at Oslo University Hospital (OUS) or Neonatal Intensive Care Units (NICUs) at OUS and Haukeland University Hospital (HUS) in Norway was undertaken from 22nd of September 2022 to 24th of November 2023. Capture of clinical data took place prior to sample analyses, and data collectors were blinded with respect to results of the laboratory tests. Outcome assessors were blinded with respect to patients' ID and clinical characteristics but not to study centre and to use of medications that could be determined by the test. We aimed to recruit 120 infants born at term and 120 born preterm (24).

### Eligibility

Infants aged 0–4 weeks were included in the study. Exclusion criteria were: Suspected adrenal disorders, critical or immediately life-threatening conditions, such as the first two days of sepsis treated with intravenous antibiotics, intraventricular haemorrhage grade 3–4, necrotizing enterocolitis, surgery within the past 5 days, malformations that interfere with sampling and weight ≤ 1 kg (criterion since 30th of September 2023 due to one serious adverse event deemed not related to study participation). Study nurses conducted regular pre-screenings of in-patient lists, and when approved by the study physicians, provided information about the study to the parent(s) of eligible infants. Whenever feasible, efforts were made to schedule sample collection at least 48 h post-birth, 2 h after potentially unpleasant medical procedures, if any, and 20 min since the last oral intake of food, medications or regurgitation/vomiting.

### Data and sample collection

Demographic and clinical data collected from the electronic patient journals (EPJ) along with information regarding the sampling session was registered in an electronic case report form (Viedoc Technologies, Uppsala, Sweden) by the study nurses.

Saliva samples were collected in the morning (0700 h to 0900 h) and/or evening (2100 h to 2300 h) using SalivaBio Infant's Swab (Salimetrics, LLC, PA, USA) according to the manufacturer's instructions and frozen in Swab Storage Tubes (Salimetrics) at −20 °C within 90 min. The batches of samples were shipped to the Hormone Laboratory either on frozen elements using an inter-laboratory transfer (transport time <12 h) or on dry ice with a commercial inter-city carrier. Upon arrival, the samples were stored at −80 °C until analysis. To retrieve saliva, the swabs were thawed at room temperature for 30–60 min and centrifuged unopened in Swab Storage Tubes at 1500 g for 15 min at room temperature in swing-out rotor, followed by discard of swab basket with swab and recapping the tube.

### Hormone measurements

Measurement of adrenal steroids, betamethasone and dexamethasone in salivary samples was performed as previously described (21). The median time between sample collection and analysis was one month, the maximal storage time did not exceed 103 days.

The analytical method (21) was validated for 50 µL saliva, but 70% of specimens had a smaller sample volume. Because accuracy had been investigated for lower volumes (21), we chose to analyse all the samples ≥1 µl correcting the concentrations of the analytes with dilution factors. The lower limit of quantitation (LLoQ) was defined as the lowest concentration giving accuracy within 20% and precision <20% in a spiked blank matrix. The number of samples with concentrations below LLoQ is presented in Table 1 along with thresholds that have been previously reported (21). Concentrations below LLoQ were calculated using calibration curves extended below LLoQ by forcing the calibration curves through zero but only if qualitatively approved peak shape, correct ion-ratio and acceptable signal-to-noise ratio (at least > 3:1) were present. Measurements < LLoQ that failed to satisfy these criteria were considered to be lower than the limit of detection (LoD).

**Table 1:** Lower limit of quantification (LLoQ) and missing values for salivary adrenal steroids within the analysed samples (*n* = 412) from the new-born infants (*n* = 174).

	LLoQ for sample volumes^a^				
Analyte	5µL	10µL	50µL	Samples with missing values (%)	Samples above LLoQ (%)^b^
17OH-pregnenolone, nmol/L	35	35	4.0	1.70	0.00
17OH-progesterone, pmol/L	600	200	60	0.97	48.53
11-deoxycortisol, pmol/L	200	200	20	0.97	54.90
Cortisol, nmol/L	4.0	4.0	0.1	0.24	55.72
Cortisone, nmol/L	4.0	4.0	0.1	0.97	88.73
Deoxycorticosterone, pmol/L	200	90	15	0.97	7.60
Corticosterone, pmol/L	2000	750	150	0.97	12.25
Aldosterone, pmol/L	2500	530	100	1.21	64.13
DHEA, nmol/L	7.5	7.5	0.8	1.70	7.65
DHEAS, µmol/L	0.6	0.5	0.1	1.70	1.98
Androstenedione, pmol/L	450	400	150	6.55	43.64
Testosterone, pmol/L	400	400	40	7.77	10.79

a as previously reported in: Dahl SR, Bakke LH, Thorsby PM, Zykova SN. An LC-MS/MS assay for simultaneous determination of 13 steroid hormones and two synthetic steroids in saliva: potential utility for paediatric population and beyond. Scand J Clin Lab Invest. 2024 Nov-Dec;84(7-8):527-534. doi: 10.1080/00365513.2024.2437620. Epub 2024 Dec 18. PMID: 39696873.

b After excluding missing values.

### Definitions

Prematurity status was categorized as: Moderate to late preterm (GA 32 to ≤36 weeks+6 days), Very preterm (GA 28 to <32 weeks), or Extremely preterm (GA <28 weeks). Exposure to synthetic steroids was defined at the infant level as either pre-natal exposure to Celestone Chronodose (Organon Norway AS) and/or post-natal administration of dexamethasone, betamethasone or hydrocortisone, and/or mass-spectrometric detection of betamethasone or dexamethasone in any of the six samples. Samples from infants who received hydrocortisone (N_sample_=2) were excluded from the analyses for cortisol and cortisone due to inability of our method to differentiate between the synthetic and endogenous hormones. Exposure to sympathomimetics was defined at the sample level as either adrenaline, dopamine, dobutamine or caffeine administered within 24 h before collection of the respective sample.

### Missing values

Samples with volume <50 µL and no quantifiable signal resulted in missing values for some of the steroid hormones and were not considered in the analyses (Table 1). Missing values for the journal entries of whether betamethasone or dexamethasone was administered were replaced with “no” (four and six samples respectively), unless signal measured, then “yes” imputed (one sample each for both medications). Unknown status of pre-natal exposure to Celestone was replaced with “no” for five samples coming from two infants born at GA 36 and 38 respectively. All missing values for other exposures within the last 24 h prior to sampling were imputed as “no” (*n*_samples_=7 for hydrocortisone, other synthetic steroids, adrenaline, dopamine and dobutamine; *n*_samples_=11 for caffeine), status as sleeping (*n*_samples_=18) and status as crying at sampling (*n*_samples_=9). The latter category included infants that “rejected/extruded” swabs without crying.

### Statistical analyses

To assess the sources of biological variation for appropriate reference interval partitioning, for each hormone we constructed a linear mixed model with various factors (fixed effects) that can possibly influence measured hormone levels (log-transformed). Samples with measured concentration below LLoQ were not excluded from the analyses to avoid bias to the right in reference intervals as well as underrepresentation of normal samples with low concentrations of steroid hormones in our models. Regression analyses were initially performed on and reported as data from all available samples controlling for specimen volume input (categorised as 50 µL, 40–49 µL, 30–39 µL, 20–29 µL, 10–19 µL or 1–9 µL), followed by sensitivity analyses (data not shown) with volumes above the highest category giving statistically significant effects. Only effects shown as significant in that second step were used for the reference interval partitioning. Function *lmer* from R package *lme4*, version 1.1–35.3, was used.

The following fixed effects were explored in the models (note that number of samples for each subcategory is reported before volume-based filtering and discarding the samples with absent measurements for each hormone, hence numbers in each hormone model may vary):

- Time of day for specimen collection: morning (*n* = 241) or evening (*n* = 171);
- Hours since birth: ≤48 h (*n* = 103) or >48 h (*n* = 309);
- Gestational age at birth: ≥37 weeks (*n* = 154), 32 to <37 weeks (*n* = 187), 28 to <32 weeks (*n* = 49), <28 weeks (*n* = 22);
- Delivery mode: vaginal (*n* = 205) or caesarean (*n* = 207);
- Apgar score at 1 min after birth: continuous, from 1 to 10;
- Sex: female (*n* = 193) or male (*n* = 219);
- Highest grade of respiratory support received 24 h prior to sampling: 1) none (*n* = 300), 2) high-flow nasal cannula (*n* = 47), 3) continuous positive airway pressure (CPAP) (*n* = 59) or 4) mechanical ventilation (*n* = 6);
- Perinatal exposure to synthetic steroids: no (*n* = 216) or yes (*n* = 196);
- Exposure to sympathomimetics 24 h prior to sampling: no (*n* = 286) or yes (*n* = 126);
- Sleeping during sampling: no (*n* = 218) or yes (*n* = 194);
- Crying during sampling: no (*n* = 378) or yes (*n* = 38).

Random effects controlled for were participant's study ID (*n* = 174 subjects) and date of hormone assay (15 batches).

We calculated reference intervals using parametric and robust methods following recommendations of Horn et al. (25), both on log-transformed values, with reference intervals transformed back to the original scale.

### Ethics

The study was approved by the Regional Ethical Research Authority (reference: 247285), the Data Protection Officer (reference: 21/10703) and relevant institutional authorities at OUS and HUS (reference numbers 334217 and 4061, respectively). Written informed consent from both parents (when two) was obtained for all the study participants.

## Results

### Demography and clinical characteristics

**Figure 2:**
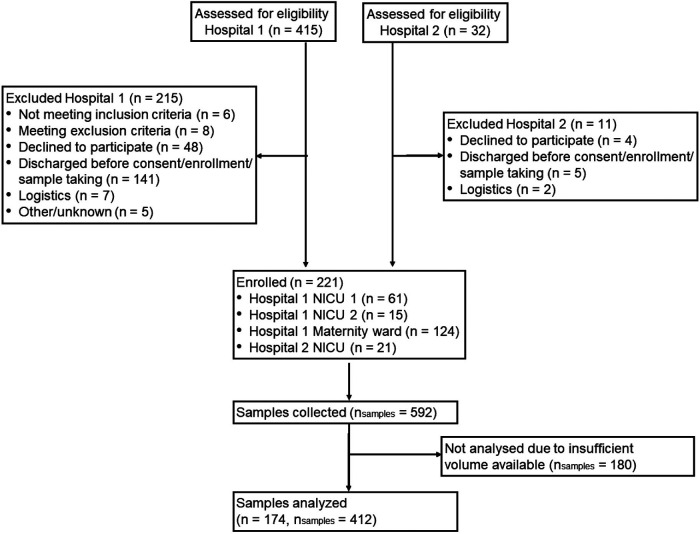
Flow chart of inclusion.

A total of 447 infants were assessed for eligibility (Figure 2). Of these, 226 infants were excluded, the majority due to infant discharge prior to sampling. From the 221 infants enrolled, a total of 592 samples were collected. However, 180 samples had insufficient volume available (<1 µL), leading to the analysis of steroid hormones in 412 specimens. Anthropometric and clinical characteristics of the infants included in the analyses are summarized in Tables 2, 3.

**Table 2:** Clinical characteristics of the 174 study participants.

	Gestational age at birth (weeks)			
Characteristic	<28	28 to <32	32 to <37	≥37
Number of infants, *n* (%)	5 (3%)	13 (7%)	52 (30%)	104 (60%)
Female, *n* (%)	1 (0.6%)	2 (1.1%)	26 (15%)	56 (32%)
Age at 1st sample taking in days, median (25th-75th %tile)	15 (1–28)	10 (8–14)	4 (1–10)	2 (1–3)
Weight at 1st sample taking in grams, median (25th-75th %tile)	1100 (975–1120)	1470 (1380–1591)	2030 (1772–2522)	3385 (3119–3767)
Apgar score at 1 min, median (25th-75th %tile)	6 (1–7)	8 (5–8)	8 (7–9)	9 (8–9)
Caesarian delivery, *n* (%)	1 (0.6%)	8 (4.6%)	30 (17%)	33 (19%)
Peri-natal exposure to synthetic steroids, *n* (%)	5 (3%)	12 (7%)	29 (17%)	5 (3%)

**Table 3:** Characteristics of the 412 samples analysed.

	Gestational age at birth (weeks)			
Characteristic	<28	28 to <32	32 to <37	≥37
Saliva samples, *n* (%)	22	49	187	154
Total saliva volume (µL) obtained, median, (25th-75th %tile)	47.5 (5–90)	25 (10–65)	35 (11–85)	34 (11–90)
Highest grade of respiratory support in the last 24h:				
• nasal cannula, *n* (%)	0	18 (4.4%)	25 (6.1%)	4 (1%)
• CPAP, *n* (%)	17 (4.1%)	12 (2.9%)	25 (6.1%)	5 (1.2%)
• mechanical ventilation, *n* (%)	4 (1%)	0	2 (0.5%)	0
Sympathomimetics (incl. caffeine) 24 h prior to sampling, *n* (%)	21 (5.1%)	42 (10%)	58 (14%)	5 (1.2%)
Intravenous lines up to 24 h prior to any sampling event, *n* (%)	13 (3.2%)	13 (3.2%)	66 (16%)	13 (3.2%)
Last feeding being nasogastric, *n* (%)	18 (4.4%)	40 (10%)	81 (20%)	5 (1.2%)

### Adrenal steroids, infant characteristics and specimen collection settings

The exploratory analyses of factors with relevance for biological variation in adrenal steroids are presented separately for glucocorticoids, mineralocorticoids and androgens (Tables 4A–C, respectively). In line with CAH being an exclusion criterion, 21-deoxycortisol was below detection threshold in all the samples analysed and therefore omitted from statistical analyses.

**Table 4A:** Linear mixed models for concentration of glucocorticoids in saliva of infants aged 0–4 weeks.

Predictors	17OH-pregnenolone			17OH-progesterone			11-deoxycortisol			Cortisol			Cortisone		
	Change in %	CI	*p*	Change in %	CI	*p*	Change in %	CI	*p*	Change in %	CI	*p*	Change in %	CI	*p*
Time: evening	−0.2	−12.9–14.4	0.977	6.3	−6.7–21.2	0.358	5.8	−4.6–17.4	0.282	1.4	−16.7–23.3	0.892	8.0	−1.1–17.9	0.087
Hours since birth: >48	−20.3	−34.1 – −3.5	**0**.**020**	−23.5	−38.1 – −5.4	**0**.**014**	−18.7	−32.6 – −1.9	**0**.**031**	−38.9	−54.2 – −18.5	**0**.**001**	−27.8	−37.3 – −16.9	**<0**.**001**
GA: 32 to <37 w.	15.4	−9.1–46.5	0.239	100.6	46.4–174.8	**<0**.**001**	100.2	46.6–173.4	**<0**.**001**	−18.6	−43.6–17.5	0.272	0.1	−18.0–22.3	0.988
GA: 28 to <32 w.	10.8	−28.3–71.1	0.643	172.1	54.7–378.6	**0**.**001**	226.6	87.2–469.8	**<0**.**001**	−29.6	−63.2–34.7	0.288	−9.4	−36.6–29.5	0.586
GA: <28 w.	16.2	−34.8–107.0	0.609	1058.5	433.8–2414	**<0**.**001**	756.7	286.0–1801	**<0**.**001**	75.9	−30.2–343.5	0.230	33.6	−19.3–121.0	0.259
Apgar at 1 min.	−0.3	−5.7–5.4	0.923	6.3	−1.4–14.6	0.110	−0.9	−8.4–7.2	0.816	2.3	−6.4–11.9	0.614	1.2	−3.7–6.2	0.640
Caesarian delivery	−19.6	−32.1 – −4.7	**0**.**012**	0.1	−19.8–24.9	0.995	20.8	−4.2–52.3	0.110	3.5	−21.1–35.8	0.803	−0.5	−13.9–15.1	0.950
Sex: male	−12.2	−26.3–4.6	0.145	31.3	5.1–64.2	**0**.**017**	−20.4	−36.7 – −0.1	**0**.**049**	12.7	−14.2–47.9	0.388	8.5	−6.1–25.4	0.269
Max respiratory support last 24h															
• Nasal cannula	37.6	1.8–86.1	**0**.**038**	143.7	73.4–242.6	**<0**.**001**	98.7	47.2–168.3	**<0**.**001**	33.9	−14.2–108.9	0.198	51.8	21.2–90.0	**<0**.**001**
• CPAP	36.1	0.8–83.8	**0**.**044**	46.1	3.1–107.0	**0**.**033**	72.5	26.9–134.3	**0**.**001**	152.3	60.9–295.5	**<0**.**001**	66.9	32.9–109.4	**<0**.**001**
• Mechanical ventilation	−20.1	−58.8–55.1	0.507	35.2	−32.1–169.4	0.390	62.1	−8.1–185.9	0.095	162.5	−14.9–709.6	0.093	62.8	−3.4–174.4	0.067
Synthetic steroids: yes	2.6	−19.1–30.3	0.830	32.0	−3.9–81.3	0.086	5.8	−23.5–46.5	0.732	−32.5	−53.5 – −1.9	**0**.**039**	−15.6	−31.2–3.6	0.104
Sympatomimetics: yes	12.7	−12.3–44.9	0.350	−12.0	−33.5–16.4	0.369	−7.9	−27.4–16.8	0.497	4.4	−28.0–51.3	0.819	−4.0	−20.1–15.3	0.662
Sleeping: yes	0.6	−13.3–16.9	0.933	2.0	−12.3–18.6	0.799	−8.9	−19.6–3.1	0.137	0.9	−19.0–25.6	0.936	−6.0	−15.0–4.0	0.232
Crying: yes	−24.2	−41.2 – −2.5	**0**.**031**	−19.3	−38.0–5.0	0.110	7.4	−14.1–34.2	0.531	−3.2	−33.2–40.3	0.864	−2.9	−18.5–15.8	0.746
Sample volume: 40–49 µL	36.9	1.3–85.0	**0**.**041**	16.0	−14.2–56.9	0.334	4.1	−18.6–33.2	0.746	11.1	−28.8–73.1	0.643	2.9	−16.0–25.9	0.785
Sample volume: 30–39 µL	55.6	12.9–114.4	**0**.**007**	12.4	−18.5–55.0	0.476	5.0	−19.2–36.5	0.712	17.7	−26.6–88.7	0.498	6.5	−14.1–32.1	0.564
Sample volume: 20–29 µL	58.6	15.2–118.3	**0**.**005**	37.2	0.3–87.8	**0**.**048**	8.1	−16.1–39.2	0.548	6.2	−32.2–66.6	0.791	5.3	−14.6–29.7	0.630
Sample volume: 10–19 µL	203.0	147.7–270.6	**<0**.**001**	50.1	22.2–84.5	**<0**.**001**	7.9	−8.9–27.7	0.378	25.8	−6.1–68.6	0.124	14.5	−0.2–31.2	0.053
Sample volume: 1–9 µL	632.4	497.2–798.2	**<0**.**001**	138.9	93.7–194.7	**<0**.**001**	24.0	4.4–47.4	**0**.**015**	26.6	−6.4–71.0	0.125	11.6	−2.9–28.4	0.122
Random Effects															
*σ* ^2^	0.40			0.35			0.22			0.85			0.16		
*τ*_00_	0.07 Subject			0.27 Subject			0.40 Subject			0.27 Subject			0.11 Subject		
	0.30 Batch			0.25 Batch			0.01 Batch			0.03 Batch			0.02 Batch		
ICC	0.47			0.60			0.65			0.26			0.46		
N	172 Subjects			173 Subjects			173 Subjects			174 Subjects			173 Subjects		
	12 Batches			13 Batches			13 Batches			14 Batches			13 Batches		
Observations	404			407			407			410			407		
Marginal R^2^/Conditional R^2^	0.441/0.706			0.424/0.768			0.445/0.807			0.190/0.408			0.211/0.572		

For each predictor we report percentage change of the predictor's geometric mean hormone concentration compared to baseline's (intercept) geometric mean ((x_1_ * x_2_ * … * x_n_)^(1/n)). The intercept itself is omitted from the table. In each model, outcome is logtransformed non-missing non-zero values of the hormone concentration. As hormones have different number of missing values, number of samples and hence batches varies slightly from model to model. GA: gestational age at birth (in weeks), CPAP, continuous positive airway pressure; Max resp. support, highest grade of respiratory support received during last 24 h before saliva sampling; w., weeks.

**Table 4B:** Linear mixed models for concentration of mineralocorticoids in saliva of infants aged 0–4 weeks.

Predictors	Deoxycorticosterone			Corticosterone			Aldosterone		
	Change in %	CI	*p*	Change in %	CI	*p*	Change in %	CI	*p*
Time: evening	1.5	−10.8–15.4	0.823	13.4	−6.3–37.2	0.197	−5.9	−16.6–6.2	0.321
Hours since birth: >48	−11.2	−26.0–6.6	0.203	−15.7	−35.3–9.6	0.202	−18.6	−34.6–1.4	0.066
GA: 32 to <37 w.	23.2	−2.2–55.1	0.076	72.8	25.5–137.8	**0**.**001**	23.7	−13.6–77.3	0.245
GA: 28 to <32 w.	27.8	−15.5–93.2	0.245	65.5	−6.0–191.3	0.081	90.9	0.5–262.5	**0**.**048**
GA: <28 w.	79.6	2.8–213.8	**0**.**040**	238.3	54.6–640.3	**0**.**002**	99.3	−20.0–396.4	0.138
Apgar at 1 min.	−3.7	−8.7–1.7	0.174	3.4	−4.3–11.7	0.400	−3.7	−12.0–5.3	0.406
Caesarian delivery	5.1	−10.8–23.8	0.551	−14.0	−32.1–9.1	0.213	−2.8	−25.4–26.6	0.833
Sex: male	−4.2	−19.0–13.3	0.617	8.9	−14.4–38.5	0.487	8.9	−16.1–41.3	0.522
Max respiratory support last 24h									
• Nasal cannula	8.8	−18.1–44.4	0.561	20.2	−20.5–81.6	0.382	−27.7	−48.9–2.3	0.067
• CPAP	26.2	−5.4–68.2	0.113	107.7	38.4–211.5	**<0**.**001**	36.7	−4.2–95.1	0.085
• Mechanical ventilation	191.5	55.0–448.0	**0**.**001**	101.8	−20.7–413.5	0.140	3.7	−46.4–100.6	0.915
Synthetic steroids: yes	−6.6	−25.8–17.5	0.558	−16.4	−39.6–15.5	0.276	−2.6	−32.9–41.2	0.887
Sympatomimetics: yes	−7.6	−27.2–17.2	0.513	−19.6	−42.7–12.7	0.205	−0.0	−24.1–31.8	0.999
Sleeping: yes	−2.2	−15.1–12.7	0.759	−7.9	−25.4–13.6	0.439	−8.5	−20.8–5.7	0.225
Crying: yes	3.2	−18.8–31.3	0.794	−18.6	−43.1–16.5	0.260	0.7	−22.4–30.7	0.957
Sample volume: 40–49 µL	16.1	−12.7–54.5	0.303	75.9	14.9–169.5	**0**.**010**	−1.2	−25.8–31.6	0.934
Sample volume: 30–39 µL	33.0	−1.9–80.1	0.066	22.2	−22.3–92.2	0.385	0.5	−25.9–36.3	0.973
Sample volume: 20–29 µL	81.8	35.3–144.4	**<0**.**001**	67.9	8.6–159.7	**0**.**020**	6.3	−20.9–42.8	0.686
Sample volume: 10–19 µL	183.8	134.6–243.4	**<0**.**001**	160.5	97.3–243.9	**<0**.**001**	8.4	−11.0–32.2	0.421
Sample volume: 1–9 µL	519.0	410.0–651.4	**<0**.**001**	374.4	256.8–530.9	**<0**.**001**	18.0	−3.6–44.3	0.108
Random Effects									
σ2	0.40			0.83			0.29		
τ_00_	0.06 Subject			0.14 Subject			0.51 Subject		
	0.15Batch			0.02 Batch			0.03 Batch		
ICC	0.34			0.17			0.65		
N	173 Subjects			173 Subjects			173 Subjects		
	13 Batches			13 Batches			13 Batches		
Observations	407			407			406		
Marginal R^2^/Conditional R^2^	0.461/0.679			0.328/0.440			0.120/0.688		

For each predictor we report percentage change of the predictor's geometric mean hormone concentration compared to baseline's (intercept) geometric mean ((x_1_ * x_2_ * … * x_n_)^(1/n)). The intercept itself is omitted from the table. In each model, outcome is logtransformed non-missing non-zero values of the hormone concentration. As hormones have different number of missing values, number of samples and hence batches varies slightly from model to model. GA: gestational age at birth (in weeks), CPAP, continuous positive airway pressure; Max resp. support, highest grade of respiratory support received during last 24 h before saliva sampling; w., weeks.

**Table 4C:** Linear mixed models for concentration of androgens in saliva of infants aged 0–4 weeks.

Predictors	DHEAS			DHEA			Androstenedione			Testosterone		
	Change in %	CI	*p*	Change in %	CI	*p*	Change in %	CI	*p*	Change in %	CI	*p*
Time: evening	11.8	−3.0–28.9	0.123	8.9	−5.8–25.9	0.249	2.4	−9.7–16.1	0.712	11.8	−3.8–30.0	0.144
Hours since birth: >48	−39.1	−52.0 – −22.6	**<0**.**001**	−8.1	−25.3–13.0	0.421	−20.2	−34.7 – −2.5	**0**.**027**	−26.6	−41.4 – −8.2	**0**.**007**
GA: 32 to <37 w.	5.2	−27.1–51.9	0.785	24.3	−4.4–61.5	0.104	24.1	−7.6–66.5	0.151	16.9	−14.3–59.4	0.323
GA: 28 to <32 w.	−0.2	−48.8–94.7	0.996	76.3	9.9–183.0	**0**.**019**	22.1	−28.6–108.5	0.465	−5.2	−46.1–66.6	0.851
GA: <28 w.	269.6	48.8–818.4	**0**.**005**	434.6	181.6–914.9	**<0**.**001**	497.5	191.5 – 1124.6	**<0**.**001**	340.5	109.2 – 827.7	**<0**.**001**
Apgar at 1 min.	3.4	−5.4–12.9	0.462	0.0	−6.0–6.5	0.991	5.9	−1.4–13.7	0.115	1.6	−5.6–9.3	0.678
Caesarian delivery	10.1	−15.2–43.0	0.469	−1.9	−18.9–18.6	0.842	2.8	−17.0–27.5	0.798	−7.4	−25.9–15.7	0.497
Sex: male	17.7	−9.3–52.9	0.219	−9.8	−25.6–9.5	0.298	9.7	−11.2–35.5	0.389	85.9	48.9–132.0	**<0**.**001**
Max respiratory support last 24h												
• Nasal cannula	51.8	2.8–124.0	**0**.**036**	17.4	−15.8–63.6	0.343	56.5	11.9–118.9	**0**.**009**	43.0	−1.4–107.5	0.059
• CPAP	13.4	−23.4–67.9	0.529	2.4	−26.0–41.7	0.887	26.6	−8.2–74.8	0.150	31.5	−7.9–87.6	0.131
• Mechanical ventilation	25.2	−41.4–167.2	0.561	134.5	14.2–381.6	**0**.**020**	76.2	−7.4–235.1	0.084	96.7	−6.3–313.1	0.074
Synthetic steroids: yes	22.6	−15.5–78.0	0.283	21.3	−6.9–58.2	0.153	8.2	−20.5–47.1	0.617	26.8	−7.8–74.6	0.144
Sympatomimetics: yes	12.6	−17.6–53.9	0.457	−10.5	−31.7–17.4	0.422	−9.8	−31.0–17.8	0.446	−4.5	−30.2–30.7	0.775
Sleeping: yes	−3.6	−18.4–13.9	0.669	−16.1	−28.6 – −1.5	**0**.**033**	−3.3	−16.5–11.8	0.647	25.1	5.4–48.4	**0**.**011**
Crying: yes	−20.2	−40.5–6.9	0.130	−20.3	−39.4–4.9	0.106	−11.4	−31.6–14.7	0.356	−8.7	−32.1–22.7	0.546
Sample volume: 40–49 µL	9.0	−21.8–51.9	0.610	23.2	−11.0–70.5	0.208	−12.6	−34.1–16.1	0.352	11.9	−19.6–55.6	0.505
Sample volume: 30–39 µL	23.1	−13.5–75.0	0.248	53.4	8.6–116.6	**0**.**015**	4.3	−23.4–42.2	0.788	11.0	−22.8–59.6	0.572
Sample volume: 20–29 µL	44.2	1.5–104.7	**0**.**041**	53.1	8.6–115.9	**0**.**015**	29.4	−6.0–78.2	0.113	28.3	−11.7–86.5	0.190
Sample volume: 10–19 µL	61.0	28.4–102.0	**<0**.**001**	189.8	133.7–259.4	**<0**.**001**	35.1	11.1–64.2	**0**.**003**	155.5	103.5–220.7	**<0**.**001**
Sample volume: 1–9 µL	187.5	128.2–262.3	**<0**.**001**	604.5	465.6–777.7	**<0**.**001**	128.4	87.1–178.8	**<0**.**001**	318.0	231.6–427.0	**<0**.**001**
Random Effects												
σ2	0.41			0.46			0.31			0.44		
τ_00_	0.41 Subject			0.11 Subject			0.23 Subject			0.20 Subject		
	0.16 Batch			0.05 Batch			0.04 Batch			0.10 Batch		
ICC	0.58			0.26			0.47			0.40		
N	172 Subjects			172 Subjects			165 Subjects			165 Subjects		
	12 Batches			12 Batches			11 Batches			10 Batches		
Observations	404			404			384			379		
Marginal R^2^/Conditional R^2^	0.260/0.690			0.520/0.644			0.336/0.648			0.478/0.687		

For each predictor we report percentage change of the predictor's geometric mean hormone concentration compared to baseline's (intercept) geometric mean ((x_1_ * x_2_ * … * x_n_)^(1/n)). The intercept itself is omitted from the table. In each model, outcome is log-transformed non-missing non-zero values of the hormone concentration. As hormones have different number of missing values, number of samples and hence batches varies slightly from model to model. GA: gestational age at birth (in weeks), CPAP, continuous positive airway pressure; Max resp. support, highest grade of respiratory support received during last 24 h before saliva sampling; w., weeks.

#### Glucocorticoids

Recent birth was associated with significantly higher concentrations of salivary 17OH-pregnenolone, 17OH-progesterone, 11-deoxycortisol, cortisol and cortisone: in samples collected more than 48 h after birth compared to samples collected less than 48 h after birth, there was a decrease by (respectively) 20%, *p* = 0.02, 24%, *p* = 0.014, 19%, *p* = 0.031, 39%, *p* = 0.001% and 28%, *p* < 0.001 (Table 4A). Gestational age at birth was associated with gradual changes in 17OH-progesterone concentrations, which were elevated by 1060**%** in extremely preterm, by 172% in very preterm and by 101% in moderately to late preterm compared to term-born neonates (*p* ≤ 0.001 for all). A similar significant trend was observed for 11-deoxycortisol where concentrations fell with progressing maturity from 757% elevation above the level of term-born infants in extremely preterm, to 227% and 100% increase in very preterm and moderate to late preterm, respectively (*p* < 0.001 for all). No such differences in 17OH-pregnenolone, cortisol and cortisone were observed.

Perinatal synthetic steroids were linked to 33% suppression in salivary free - i.e., biologically active - cortisol compared to the level in the unexposed infants (*p* = 0.039).

Respiratory support with CPAP was associated with higher concentrations of both mature glucocorticoids (152% increase in cortisol, *p* < 0.001% and 67% increase in cortisone, *p* < 0.001) and their precursors (73% increase in 11-deoxycortisol, *p* = 0.001, 46% increase in 17OH-progesterone, *p* = 0.033, and 36% increase in 17OH-pregnenolone, *p* = 0.044) compared to infants without respiratory support in models adjusted for other factors, including GA. Similar results were noted for high-flow nasal cannula which was associated with a 52% increase in cortisone (no significant results for cortisol), and more pronounced elevation of precursors with 99% increase in 11-deoxycortisol, 144% increase in 17OH-progesterone, *p* < 0.001 for both, and a 38% increase in 17OH-pregnenolone, *p* = 0.038. Infants who cried during specimen collection had 24% lower 17OH-pregnenolone concentrations (*p* = 0.031).

Male sex was associated with 31% higher 17OH-progesterone (*p* = 0.017) and 20% lower 11-deoxycortisol (*p* = 0.049).

#### Mineralocorticoids

Analysis of mineralocorticoids (Table 4B) revealed no significant factors associated with aldosterone levels, other than being born very pre-term (91% increase compared to those born at term, *p* = 0.048)**.** The effect of GA at birth was also present for corticosterone, with an increase of 238% (*p* = 0.002) for infants born before GA 28 weeks and an increase of 73% (*p* = 0.001) for infants born between GA 32 and 37 weeks compared to those born at term. The effect of birth between GA 28 and 32 weeks did not reach statistical significance.

Being on mechanical ventilation was associated with a 192% increase in deoxycorticosterone (*p* = 0.001), while receiving respiratory support with CPAP was associated with a 108% increase in corticosterone (*p* < 0.001).

We neither expected nor observed any effect of sex or synthetic steroids on mineralocorticoids.

#### Androgens

As previously reported for serum (26), the main products of the foetal zone of the neonatal adrenals, DHEA and DHEAS (Table 4C), were significantly higher in infants born before GA 28 weeks than in the term-born infants (by 435%, *p* < 0.001% and 270%, *p* = 0.005, respectively). The same pattern was observed for the more mature androgens androstenedione (by 498%, *p* < 0.001) and testosterone (by 341%, *p* < 0.001). We did not observe significant associations of other term categories except 76% elevation in DHEA (*p* = 0.019) in very preterm infants. DHEA, DHEAS and androstenedione did not differ between the sexes, while testosterone was significantly higher in boys (by 86%, *p* < 0.001).

Respiratory support was linked with changes in several androgens: high-flow nasal cannula was associated with an increase in DHEAS and androstenedione concentrations (52%, *p* = 0.036% and 57%, *p* = 0.009 respectively), while mechanical ventilation was associated with a 135% increase (*p* = 0.02) in DHEA.

We observed 16% lower DHEA (*p* = 0.033) and 25% higher testosterone (*p* = 0.011) in infants that were sleeping.

#### Low specimen volume and concentration of adrenal steroids

Cortisol, cortisone and aldosterone concentrations in saliva had no association with volume of specimen processed for steroid analyses (Table 4A–C). Other adrenal steroids, however, showed increasing concentrations in saliva with decreasing sample volume. This effect was proportionate to degree of analyte hydrophobicity.

#### Synthetic steroids

**Figure 3:**
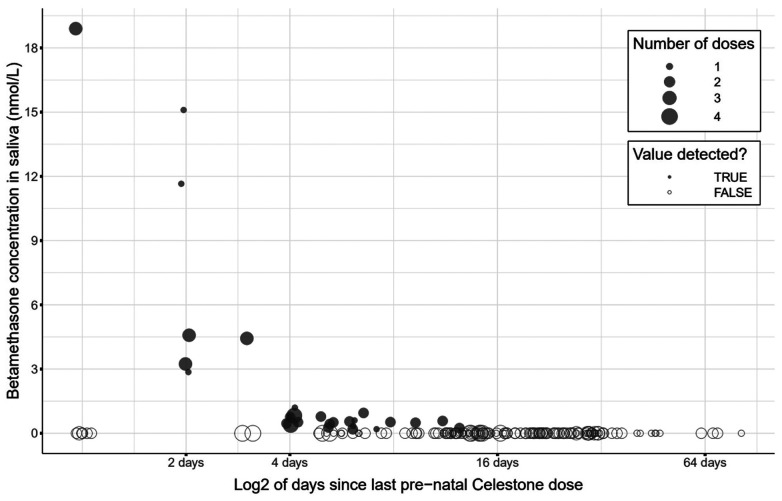
Concentration of betamethasone in saliva of infants with prenatal exposure to CelestoneChronodose®. Concentration of betamethasone was determined using LC-MS/MS in salivary samples from new-born infants who had been exposed to CelestoneChronodose® prenatally (n = 50) to accelerate lung maturation. The drug (12 mg betamethasone per dose, 6 mg/ml injection fluid with 3.0 mg/ml of betamethasone acetate, equivalent to 2.71 mg/ml of betamethasone, and 3.945 mg/ml of betamethasone sodium phosphate, equivalent to 3.0 mg/ml of betamethasone) was administered intramuscularly to infants' mothers due to threatening premature delivery, typically as two doses given 24 hours apart, eventually followed by two more doses if still pregnant after more than two weeks. Small random shift (10% jitter) was added to x-coordinates to reduce overlay and improve readability of the plots.

Betamethasone administered to pregnant women at risk for premature delivery could be detected in saliva of their newborn children up to 12 days following the last injection (Figure 3). There was a rapid decline in betamethasone concentration in the infant's saliva, with an increasing number of days between Celestone injection to the mother and the sampling event. We could also detect dexamethasone (mean 1.0 nmol/L, range 0.13–3.8 nmol/L) in 7 of 10 infants exposed to the drug (data not shown).

### Reference intervals

Table 5 presents reference intervals calculated with the recommended robust method using median value of the available samples from each individual. For each hormone, we assessed the threshold saliva volume that did not have influence on the hormone concentrations (Table 4) and used samples above that volume for RI construction. Partitioning was based on effects identified as significant during regression analyses on the volume-based-filtered subsets of data and clinical considerations. In the Supplementary file, we in addition report calculations done with the parametric method and those based on either the highest concentration among biological replicates (to accommodate for the extremely stressful life experience the neonatal period can present) or all the available samples.

**Table 5:** Reference intervals (RI) for adrenal steroids in saliva of infants aged 0–4 weeks.

Analyte (concentration units)	N^a^	Reference intervals^b^	90% Confidence intervals	
			Lower Limit	Upper Limit
**17OH-pregnenolone** (nmol/L)				
All infants	60	<4.0	0.14–3.63	2.95–5.08
Hours since birth: >48	47	<4.0	0.1–3.5	2.6–5.2
**17OH-progesterone** (pmol/L)				
All infants	81	<2521	8.7–18.5	1697–3763
Hours since birth: >48	64	<2848	8–19.3	1809–4533
**11-deoxycortisol** (pmol/L)				
All infants	146	24–987	19.3–29.8	797–1225
GA: ≥37 w.	83	22–477	17.1–27.3	378–616
GA: 32 to <37 w.	46	53–1237	37–81	914–1825
Not on respiratory support	121	22–706	17–28	553–889
**21-deoxycortisol** (pmol/L)				
All infants	174	<200^c^		
**Cortisol** (nmol/L)				
All infants	174	0.65–39	0.52–0.81	31–50
No synthetic steroids	124	0.79–40	0.62–1.0	31–53
Synthetic steroids	51	0.40–35	0.25–0.62	21–57
Hours since birth: ≤48	74	0.91–56	0.65–1.2	39–82
Hours since birth: >48	120	0.57–28	0.44–0.73	21–36
Not on respiratory support	148	0.63–37	0.50–0.79	29–48
**Cortisone** (nmol/L)				
All infants	173	18–154	16–20	138–174
Hours since birth: ≤48	74	24–222	19–30	184–271
Hours since birth: >48	119	17–127	15–19	112–146
Not on respiratory support	148	17–149	15–20	131–169
**Deoxycorticosterone** (pmol/L)				
All infants	73	<39	2.1–3.3	31–49
Hours since birth: >48	58	<43	2.1–3.6	32–59
**Corticosterone** (pmol/L)				
All infants	72	<763	8.6–18	486–1134
**Aldosterone** (pmol/L)				
All infants	164	212–4202	175–249	3664–4928
**DHEA** (nmol/L)				
All infants	72	<2.4	0.07–0.12	1.7–3.2
**DHEAS** (µmol/L)				
All infants	80	<0.10	0.002–0.004	0.07–0.15
Hours since birth: >48	63	<0.10	0.003–0.007	0.1–0.36
Girls	41	<0.10	0.002–0.005	0.06–0.13
Boys	39	<0.14	0.002–0.004	0.08–0.26
**Androstenedione** (pmol/L)				
All infants	82	<1515	25–46	1079–2096
Not on respiratory support	61	<1004	28–49	746–1366
**Testosterone** (pmol/L)				
All infants	82	<201	2.3–4.7	135–300
Hours since birth: >48	63	<153	2.8–5.7	104–232
Girls	41	<104	2.3–4.7	70–169
Boys	41	<317	3.1–7.3	182–588

a For each hormone, we assessed the threshold saliva volume that does not have influence on the hormone concentrations (Table 4) and used samples above that volume for RI construction (17OH-pregnenolone: =50 µL; 17OH-progesterone: ≥30 µL; 11-deoxycortisol: ≥10 µL; cortisol: ≥1 µL; cortisone: ≥1 µL; deoxycorticosterone**:** ≥40 µL; corticosterone: ≥40 µL; aldosterone: ≥1 µL; DHEA: ≥40 µL; DHEAS: ≥30 µL; androstenedione**:** ≥20 µL; testosterone: ≥20 µL) using robust method. We used all samples, also those below the lower limit of quantification (LLoQ), for RI construction to avoid bias to the right. Therefore, for some metabolites, the lower limits of RI with corresponding 90% confidence intervals are below LLoQ. Here RIs are computed for groups with *n* ≥ 39 according to guidelines. For groups with 20 ≤ *n* < 39, RIs are reported in Supplementary. For groups with *n* < 20, RIs are not computed. Due to a relatively small sample size and lack of studies of hormonal trends in infants, we adopted conservative approach to outlier removal (not more than two outliers were removed for some hormones).

b RI were calculated using robust method on median value of biological replicates. Partitioning of RI was based on effects identified as significant during regression analyses on the volume-based-filtered subsets of data and clinical considerations. GA, gestational age at birth; w., weeks.

c Confidence intervals not calculated as all the measurements were below detection threshold.

## Discussion

In this work we present neonatal reference intervals and explore the sources of biological variation for adrenal steroid hormones measured in saliva with a recently developed LC-MS/MS method (21).

The concentrations of salivary adrenal steroids were generally in agreement with the corresponding reference intervals for neonatal serum for 17OH-pregnenolone, 17OH-progesterone, 11-deoxycortisol, cortisone, deoxycorticosterone, aldosterone, DHEA, DHEAS and androstenedione (27, 28). As expected, salivary concentrations of cortisol and testosterone were lower than in serum, the difference being attributable to negligible amounts of the binding proteins CBG and SHBG in saliva (22). Also, concentrations of corticosterone in saliva appeared lower than in serum (27, 28). We do not know if this difference reflects the less stressful sampling procedure or other factors, such as protein-bound transfer in blood or local metabolism.

Our first major existential crisis – birth – was associated with significant elevation of the ACTH-regulated glucocorticoids and androgens in saliva, leaving the mineralocorticoids unaffected. Following findings of Iwata et al. (29), we hypothesised that for cortisol and cortisone the significant effect of the recent birth may in reality be a masked diurnal pattern and considered a model with an interaction term between time after birth and time of day for specimen collection. We did not observe a diurnal pattern in the current study (data not shown).

Inverse associations between maturity status at birth and salivary 17OH-progesterone and 11-deoxycortisol concentrations and the lack of such a trend for cortisol and cortisone observed in this study are in line with previous publications (30, 31) that related this pattern to a combination of immaturity of steroidogenic enzymes in the adrenal glands with traumatic experiences any preterm birth inevitably encompasses. Compared to infants born at term, the concentrations of salivary 17OH-progesterone and 11-deoxycortisol in extremely premature infants were increased on average by >1000% and >750% respectively. This corresponds well to the levels reported for this GA-category by others (30) measured with immunoassays in dry blood spots and plasma (32). It would be interesting to follow up previous reports (30) to investigate 17OH-progesterone and 11-deoxycortisol in different causes for preterm birth, but our study does not have sufficient power to address this issue.

The lack of association between GA and cortisol/cortisone concentrations can be a reflection of their maternal transfer and metabolism, either transplacentally [estimated for cortisol to be 20%–50% and for cortisone 88 ± 25% at birth (33)] or with milk. We could not with confidence ascertain whether steroids measured in saliva were produced by the mothers or babies. The effect of milk and formula feeding on concentration of salivary steroids should be further investigated to optimise saliva sampling procedures.

In contrast to reports of elevated postnatal 17OH-pregnenolone in plasma of premature new-borns (34), we did not observe any association between salivary 17OH-pregnenolone and GA in our models, but we rather saw an association with being on respiratory support, which is linked to both stress and prematurity. Our finding of a 20% higher concentration of salivary 17OH-pregnenolone in infants delivered vaginally compared to those with caesarean delivery is interesting and suggests a potential role of this metabolite as a marker of neonatal stress. Indeed, in the study by Hingre (32), plasma 17OH-pregnenolone along with DHEA showed the greatest elevation in response to ACTH-test in 4-day-old infants born at GA 26 ± 2 weeks. In term-born babies, scheduled caesarean delivery was associated with a 50% reduction in umbilical plasma cortisol concentration compared to both vaginally delivered infants and those born via emergency caesarean delivery, possibly reflecting a less stressful birth process in the former (35). More studies are needed to elucidate the diagnostic utility of salivary adrenal hormones as markers of neonatal stress. Excessive activation of the hypothalamic-pituitary-adrenal axis in early life has been suggested to be the mechanistic explanation for the links between low weight at birth and cardiovascular mortality later in life (36), procedural pain from skin-breaking procedures and cognitive and motor developmental delays (37), as well as many ailments and adverse outcomes of preterm born infants (38).

One of the most important hypothesis-generating findings of this study is 33% lower concentration of cortisol in combination with unaffected aldosterone in saliva from infants exposed to perinatal glucocorticoids. Such a reduction can be clinically significant. It is also likely to be underestimated: as this subgroup included fewer infants with recent exposure than those who received the drug > five half-lives previously (Figure 3). This biochemical pattern along with persistence of betamethasone in saliva from the offsprings for as long as 12 days following maternal administration suggests that the premature newborns could be at risk of developing tertiary adrenal insufficiency. Indeed, many conditions commonly seen in premature infants – such as euvolemic hyponatraemia, hypoglycaemic crises, unstable blood pressure, increased susceptibility to infection and weight loss - are also hallmark signs of iatrogenic adrenal insufficiency in adults (17). It may be that exposure to antenatal steroids might worsen these conditions by causing tertiary adrenal insufficiency, further compromising the infant's ability to regulate stress and metabolism. The levels of betamethasone in saliva were generally in agreement with previously published pharmacokinetic models (39). However, the total analytical repeatability for our method is 15%–17% for this concentration range, which according to our standards allows only semiquantitative assessment of betamethasone. More studies are needed to investigate whether there is a link between salivary concentrations of betamethasone/dexamethasone and neonatal outcomes, and to optimise dosage depending on length of gestation and maternal factors in both singleton and multiple pregnancies.

We noted higher (up to 500%) concentrations of steroid hormone precursors in saliva with decreasing sample volume. This increase was proportional to the degree of analyte hydrophobicity. We investigated and can rule out an upward bias by measuring concentrations of midrange calibrators for all the analytes in volumes of 5 and 10 µL (mean bias (±SD) for the affected metabolites were −11% (±14) and −13% (±11) respectively). There were also no systematic changes in the recovery between the analytes (100 ± 27% for all the hormones at three concentration levels each) that could be attributed to hydrophobicity (21). We therefore conclude that there were higher concentrations of the hydrophobic steroids in the oral cavity of infants with hyposalivation/dehydration compared to those from whom higher saliva volumes were obtained.

The elevated concentration of 17OH-progesterone along with reciprocal changes in 11-deoxycortisol in boys may be suggestive of a relative deficiency of CYP21A2 compared to girls which is plausible considering the need to channel more steroid precursors to testosterone production. Shunting of substrates into the androgen pathway at the expense of glucocorticoids could explain the higher risk of morbidity and mortality in premature male newborns compared to girls (40–42). Sexual dimorphism in stress responses has been attributed to several pathologies (43, 44). Therefore, we suggest further investigation of this possible effect in newborn boys, particularly the ones with iatrogenic adrenal suppression.

Being on respiratory support with CPAP or high-flow nasal cannula was associated with significant elevation of stress hormones in saliva. The lack of such an association in infants on mechanical ventilation could be explained by more efficient pain-management in this group or more effective correction of hypoxia or, most probably, too small of a sample size to capture the effect (six samples from three infants). A somewhat more immature pattern of stress response in users of high-flow nasal cannula compared to users of CPAP could be related to differences in achieved maturation, or to less efficient hypoxia correction, or more stress in the former group. We adjusted our models for maturity status and other relevant factors, but the study was not designed to fully address this issue. The impact of mechanical ventilation on deoxycorticosterone needs further exploration as all the infants in this group were born preterm. As use of respiratory support is closely linked with immaturity and illness severity, these associations should not be interpreted as independent causal effects. More research is needed to establish potential contribution of hypoxia, blood pressure medications, hydration status, electrolyte balance and the type of respiratory support to variability in salivary mineralocorticoids and stress hormones in both term-born and preterm neonates.

Simultaneous non-invasive determination of several adrenal hormones along with medications affecting steroidogenesis can hopefully aid in the work-up of patients with both prematurity and CAH. In a recent study, Lasarev et al. using principal component analysis achieved a 3-fold increase in positive predictive value of a test based on concentration of five adrenal steroids (17OH-progesterone, 11-deoxycortisol, androstenedione, 21-deoxycortisol, and cortisol) in dried blood spots compared to the standard screening approach to 21-hydroxylase deficiency (45).

### Limitations

The cross-sectional design of this work does not allow us to infer any causality between the factors studied and salivary concentrations of steroid hormones. We chose not to collect blood/serum for ethical reasons and could therefore not compare our measurements directly with corresponding reference tests. We acknowledge that some of the fixed effects in the linear mixed model used for assessing the sources of biological variation are connected, as, for example, almost all term infants received no respiratory support and almost all extremely preterm infants received either CPAP or mechanical ventilation. Also, because of the need for treatment, premature infants were more likely to experience stress, have longer hospital stays and provide more replicate samples, while the majority of infants born at term were recruited from the Maternity ward and not from NICU.

The volume of saliva was lower than desired in many samples collected in this study. This compromises the accuracy of measurements in the lower concentration range. We also acknowledge that for some predictors we do not have enough samples in all categories to make meaningful statistical conclusions. We acknowledge that for some reference intervals' partitions small sample size leads to wider than desirable confidence intervals which increases statistical uncertainty. We hope that implementation of this salivary test into clinical practice will lead to an increase of available data and subsequent improvement of reference intervals using non-direct methodology.

### Strengths

The strengths of our study are its unique not-readily accessible population, multicentre design, eligibility criteria ensuring a sample of newborns representative for NICUs including extremely preterm (born <28 weeks of gestation), EPJ-derived clinical and anthropometric information, repetitive sampling, standardised pre-analytical procedure (including morning and evening specimen collection sessions, choice and handling of swabs, recording of crying, distress and sleeping at sample taking and use of thoroughly validated sensitive methodology for hormone determination by highly trained professionals in a specialised accredited hospital laboratory.

### Recommendations for sample collection

We recommend standardising saliva collection to non-crying non-sleeping infants whenever possible. The recommended saliva sample volume is 50 µL for all hormones, but 1 µL can be acceptable for cortisol, cortisone and aldosterone, 10 µL for 11-deoxycortisol, 20 µL for androstenedione and testosterone, 30 µL for 17OH progesterone and DHEAS, and 40 µL for deoxycorticosterone and DHEA to rule out overproduction. As all 17OH-pregnenolone measurements in our study were below LLoQ (indicating an analytical variation >20% throughout normal concentration ranges), replicate samples should be taken in future studies to explore diagnostic potential of this analyte. At least two samples should also be taken for analyses of deoxycorticosterone, 17OH-progesterone, DHEA, androstenedione and betamethasone because their total analytical repeatability exceeded the cutoff of 15% being 12%–16%, 19%–22%, 16%–17%, 16%–22% and 15%–17%, respectively.

## Conclusions

The ability to assess adrenal steroid profiles in saliva of newborns, and particularly in premature infants, represents a significant advancement in neonatal medical care. This saliva-based test offers a less stressful and less invasive alternative to traditional blood tests. Not only can it improve screening, differential diagnostics and monitoring for conditions such as CAH and water and electrolyte disorders, but it also has the potential to prevent iatrogenic adrenal and immune suppression in preterm and ill neonates treated with glucocorticoids. Furthermore, the test can be applied for pain and stress detection and management in the NICU. Further studies are needed to establish the diagnostic accuracy, decision limits, clinical utility and patient benefit of this panel test.

## Data Availability

The datasets presented in this article are not readily available because permission from The Regional Health Authorities and Data Protection Officers should be obtained. Requests to access the datasets should be directed to https://rekportalen.no/ and https://www.oslo-universitetssykehus.no/en/about-oslo-university-hospital/contact-us/.
