# Supplementary material for: Neonatal reference intervals for salivary steroids: focus on prematurity, stressful events and perinatal betamethasone
Source: Front Pediatr. 2026 Jun 16;14:1808446. doi: 10.3389/fped.2026.1808446 (PMC13281276; doi:10.3389/fped.2026.1808446)

Supplementary table: Reference intervals for adrenal steroids in saliva of infants aged 0-4 weeks calculated using parametric and robust methods

(A) **17OH-pregnenolone** (nmol/L) reference intervals, based on all samples =50 µL

Groups	CI	All samples			Median of biological replicates			Highest value of biological replicates		
		N	Parametric	Robust	N	Parametric	Robust	N	Parametric	Robust
All infants		99	*0.13-4.49*	*0.13-4.84*	60	*0.14-3.63*	*0.14-3.83*	60	*0.16-4.33*	*0.15-4.66*
	Lower		*0.10-0.17*	*0.10-0.17*		*0.11-0.20*	*0.10-0.18*		*0.12-0.21*	*0.11-0.2*
	Upper		*3.48-5.81*	*3.55-6.52*		*2.70-4.90*	*2.95-5.08*		*3.19-5.89*	*3.62-6.27*
Hours since birth: ≤48		22	*0.25-5.19*	*0.23-6.36*	16			16		
	Lower		*0.16-0.40*	*0.13-0.40*						
	Upper		*3.26-8.25*	*3.81-11*						
Hours since birth: >48		77	*0.11-4.05*	*0.11-4.37*	47	*0.14-3.32*	*0.1-3.5*	47	*0.15-3.77*	*0.14-4*
	Lower		*0.08-0.15*	*0.08-0.16*		*0.1-0.19*	*0.1-0.2*		*0.11-0.21*	*0.1-0.19*
	Upper		*3.02-5.43*	*3.03-6.23*		*2.38-4.65*	*2.6-5.2*		*2.7-5.28*	*2.96-5.48*

(B) **17OH-progesterone** (pmol/L) reference intervals, based on all samples ≥30 µL

Groups	CI	All samples			Median of biological replicates			Highest value of biological replicates		
		N	Parametric	Robust	N	Parametric	Robust	N	Parametric	Robust
All infants		148	*17.5*-2792.2	*16.6*-2832.4	81	*13.9*-2421.2	*12.8*-2521.4	81	*13.8*-3179.5	*12.6*-3289

Groups	CI	All samples			Median of biological replicates			Highest value of biological replicates		
		N	Parametric	Robust	N	Parametric	Robust	N	Parametric	Robust
	Lower		*13-23.6*	*12.3-21.6*		*9.2-20.9*	*8.7-18.5*		*9-21.3*	*8.4-18.9*
	Upper		2070.6-3765.2	2126.4-3760.5		1604.8-3653	1696.5-3763		2061.6-4903.5	2140.9-5169.8
GA: ≥37 w.		44	*9.3*-602	*7.1*-600.2	33	*8.7*-710.7	*6.5*-713.8	33	*8.8*-797.5	*6.9*-875.4
	Lower		*5.9-14.5*	*4.8-11.3*		*5-15*	*4-10.7*		*5-15.5*	*4.3-11.4*
	Upper		383.3-945.4	348.2-1155.3		410-1231.9	362.2-1441.5		454.4-1399.7	474.1-1896.6
GA: 32 to <37 w		74	*38.4*-1776	*36.1*-1841.2	35	*33.8*-1856.4	*29*-1996.1	35	*38.8*-2288.2	*33.7*-2491.9
	Lower		*27.9-52.9*	*26.1-48.1*		*20.8-54.9*	*18.2-44.7*		*23.6-63.5*	*20.8-51.9*
	Upper		1290.2-2444.7	1354.7-2555.5		1142.2-3017.2	1206-3235.2		1395.7-3751.4	1518.7-4126
GA: 28 to <32 w.		17			9			9		
GA: <28 w.		13			4			4		
Hours since birth: ≤48		34	*18.4*-4718.3	*15*-6117.8	23	*13.5*-3856.1	*9.9*-5036.4	23	*12.8*-5180.8	*9.1*-6883.4
	Lower		*9.3-36.4*	*6.7-26*		*5.8-31.5*	*4.3-19.8*		*5.2-31.5*	*3.9-18.3*
	Upper		2385.6-9332	3421.9-12303.2		1656.3-8977.7	2264.9-13096.5		2112-12708.4	2967-18823.7
Hours since birth: >48		114	*17.5*-2348.5	*16.4*-2401.8	64	*13.7*-2655.3	*12.5*-2848.1	64	*14.3*-3131.3	*13.1*-3382.9
	Lower		*12.6-24.3*	*11.9-22.4*		*8.5-21.9*	*8-19.3*		*8.8-23.2*	*8-20.8*
	Upper		1690.1-3263.3	1722.2-3356.5		1655.6-4258.6	1808.5-4533.2		1931.7-5075.8	2087.9-5367.2

(C) **11-deoxycortisol** (pmol/L) reference intervals, based on all samples ≥10 µL

Groups	CI	All samples			Median of biological replicates			Highest value of biological replicates		
		N	Parametric	Robust	N	Parametric	Robust	N	Parametric	Robust
All infants		296	25.2-1330.8	25.6-1418.9	146	24.7-959.1	24-987	146	26.1-1190	25-1201.4
	Lower		21.4-29.7	21.1-30.5		19.9-30.7	19.3-29.8		20.8-32.7	19.6-31.6
	Upper		1128-1570.1	1210.1-1658.9		771.8-1191.7	796.5-1224.7		948.5-1493	947.9-1528.3
GA: ≥37 w.		111	20.3-453.5	20.6-484	83	22.5-452.8	21.7-476.8	83	25-472.7	24.4-505.6
	Lower		16.4-25.1	16.4-25.7		17.7-28.5	17.1-27.3		19.8-31.5	19-30.6
	Upper		367-560.3	393.5-600.6		357.5-573.6	377.6-615.6		375-595.7	405.6-654
GA: 32 to <37 w		134	40.2-1198.5	45.4-1461.2	46	49.6-982.6	53.2-1236.6	46	58.6-1269.9	57.7-1476.2
	Lower		32.6-49.6	34.1-59.8		36.1-68	36.6-80.8		42.4-81.2	37.8-84.1
	Upper		971.2-1479	1156.7-1855.1		716.5-1347.5	913.6-1824.5		917.4-1757.9	1062.4-2099.1
GA: 28 to <32 w.		37	72.1-2111.3	77.8-2797	13			13		
	Lower		48.5-107.4	45.3-132.4						
	Upper		1418-3143.6	1801.3-4704.2						
GA: <28 w.		14			4			4		
Max respiratory support:										
• none		215	21.3-826.5	21.8-877.3	121	22.7-697.4	21.8-706	121	25.2-788.9	24.3-801.8

Groups	CI	All samples			Median of biological replicates			Highest value of biological replicates		
		N	Parametric	Robust	N	Parametric	Robust	N	Parametric	Robust
	Lower		17.8-25.5	18-26.7		18.2-28.4	17-28.1		20.1-31.5	19.1-31.4
	Upper		691.1-988.4	729.6-1043.8		557.9-871.9	552.9-888.8		630.2-987.5	628.1-1014.7
• nasal cannula		32	108.4-1312.6	101.2-1433.8	15			15		
	Lower		79-148.7	70.3-136.6						
	Upper		956.8-1800.7	1068.4-1979.3						
• CPAP		45	131.7-1530.6	111.9-1610.8	17			17		
	Lower		101.3-171.1	81.1-147.4						
	Upper		1177.5-1989.5	1224.5-2302.3						

(D) **Cortisol** (nmol/L) reference intervals, based on all samples ≥1 µL

Groups	CI	All samples			Median of biological replicates			Highest value of biological replicates		
		N	Parametric	Robust	N	Parametric	Robust	N	Parametric	Robust
All infants		410	0.47-41	0.43-39	174	0.7-40	0.65-39	174	0.90-67	0.82-66
	Lower		0.4-0.55	0.37-0.50		0.57-0.88	0.52-0.81		0.72-1.1	0.67-1.0
	Upper		35-48	32-46		32-49	31-50		53-84	51-86
No synthetic steroids		215	0.62-46	0.57-45	124	0.88-42	0.79-40	124	0.93-64	0.84-63

Groups	CI	All samples			Median of biological replicates			Highest value of biological replicates		
		N	Parametric	Robust	N	Parametric	Robust	N	Parametric	Robust
	Lower		0.50-0.76	0.47-0.70		0.69-1.1	0.62-1.0		0.71-1.2	0.64-1.0
	Upper		37-57	36-56.63		33-53	31-53		49-84	48-85
Synthetic steroids		195	0.37-34	0.31-31.58	51	0.46-34	0.4-35	51	0.85-77	0.72-83
	Lower		0.29-0.46	0.25-0.39		0.30-0.70	0.25-0.62		0.54-1.3	0.47-1.1
	Upper		27-43	24-42		22-52	21-57		49-121	52-148
Hours since birth: ≤48		102	0.82-75	0.74-73.84	74	1.0-57	0.91-56	74	1.05-82	0.91-81
	Lower		0.6-1.1	0.54-0.98		0.74-1.5	0.65-1.2		0.73-1.5	0.64-1.3
	Upper		55-104	53-105		41-80	39-82		57-118	55-121
Hours since birth: >48		308	0.44-30	0.4-28	120	0.61-28	0.57-28	120	0.76-54	0.71-54
	Lower		0.37-0.52	0.33-0.47		0.47-0.78	0.44-0.73		0.58-1.0	0.55-0.91
	Upper		25-35	23-34		22-36	21-36		41-71	41-74
Max respiratory support:										
• none		300	0.45-34	0.41-33	148	0.68-37	0.63-37	148	0.86-54	0.82-54
	Lower		0.38-0.54	0.35-0.48		0.54-0.86	0.50-0.79		0.67-1.1	0.65-1.0
	Upper		29-41	27-40		29-47	29-48		42-69	43-70
• CPAP		58	0.73-87	0.56-81	22	0.9-120	0.47-113	22	1.55-183	1.0-258

Groups	CI	All samples			Median of biological replicates			Highest value of biological replicates		
		N	Parametric	Robust	N	Parametric	Robust	N	Parametric	Robust
	Lower		0.46-1.1	0.37-0.79		0.43-1.9	0.19-0.94		0.75-3.2	0.42-2.1
	Upper		55-136	45-136		57-252	39-296		88-378	130-827

(E) **Cortisone** (nmol/L) reference intervals based on samples ≥1 µL

Groups	CI	All samples			Median of biological replicates			Highest value of biological replicates		
		N	Parametric	Robust	N	Parametric	Robust	N	Parametric	Robust
All infants		408	14.2-155.2	14.2-157.2	173	18.2-151.6	18.1-154.2	173	21.4-182.6	21.3-186.3
	Lower		13-15.5	13-15.5		16.2-20.5	16-20.4		19.1-24.1	18.8-24
	Upper		142.6-169	145-170.4		135.1-170.2	137.7-173.9		162.5-205.2	165.5-210
Hours since birth: ≤48		103	22.5-218.5	22.7-234.3	74	23.3-200.8	24.2-221.6	74	22.8-239.6	23.7-264.2
	Lower		19.2-26.4	18.6-27.4		19.5-27.9	19.4-30.3		18.8-27.8	18.7-30.1
	Upper		186-256.5	195.7-274		167.8-240.2	183.6-271.1		197-291.5	217.3-322.7
Hours since birth: >48		305	13.7-122.6	13.8-125.1	119	16.4-121.4	16.6-127.2	119	19.1-148.7	19.6-157.7
	Lower		12.5-15	12.5-15.1		14.4-18.7	14.5-19.3		16.7-21.9	16.9-22.7
	Upper		112.1-134.2	114.8-136.1		106.5-138.5	112.4-145.6		130-170.2	137.8-182.7
Max respiratory support:										

Groups	CI	All samples			Median of biological replicates			Highest value of biological replicates		
		**N**	**Parametric**	**Robust**	**N**	**Parametric**	**Robust**	**N**	**Parametric**	**Robust**
• none		300	13.1-138.7	13-140.4	148	17.3-147.6	17-149	148	20.9-159.8	20.8-163.2
	Lower		11.9-14.4	11.8-14.4		15.2-19.6	15-19.5		18.5-23.6	18.4-23.7
	Upper		125.8-153	128.3-154.2		130.1-167.5	131.2-169		141.7-180.2	145.4-183.6
• nasal cannula		43	20.5-126.8	19.8-132.9	17			17		
	Lower		16.8-25	16.2-23.9						
	Upper		103.9-154.7	110.9-161.7						
• CPAP		59	22.1-211.2	21.5-223.3	22	23.7-245.9	22.1-284.3	22	31.7-314.6	32-384.8
	Lower		17.9-27.3	17.4-26.1		16.6-33.9	15.1-33		22.3-45	18.8-53
	Upper		171.1-260.7	183-282.8		172-351.5	193.9-481.3		221.5-446.8	230.9-642.2

(F) **Deoxycorticosterone** (pmol/L) reference intervals, based on samples ≥40 µL

Groups	CI	All samples			Median of biological replicates			Highest value of biological replicates		
		**N**	**Parametric**	**Robust**	**N**	**Parametric**	**Robust**	**N**	**Parametric**	**Robust**
All infants		125	*2.4*-45	*2.3*-45	73	*2.8*-38	*2.7*-39	73	*2.8*-51	*2.6*-50
	Lower		*2-2.9*	*1.9-2.8*		*2.2-3.5*	*2.1-3.3*		*2.2-3.6*	*2-3.2*
	Upper		38-55	37-55		31-48	31-49		40-65	38-66

Groups	CI	All samples			Median of biological replicates			Highest value of biological replicates		
		**N**	**Parametric**	**Robust**	**N**	**Parametric**	**Robust**	**N**	**Parametric**	**Robust**
Hours since birth: ≤48		30	*1.6*-35	*1.4*-36	20	*1.9*-31	*1.6*-34	20	*2.1*-39	*1.7*-42
	Lower		*1.1-2.5*	*0.87-2.1*		*1.2-2.9*	*0.88-2.4*		*1.3-3.4*	*0.86-2.8*
	Upper		23-52	23-57		20-48	20-56		24-62	24-74
Hours since birth: >48		95	*2.9*-47	*2.7*-46	58	*3.1*-44	*2.8*-43	58	*3.1*-54	*2.9*-54
	Lower		*2.4-3.6*	*2.2-3.3*		*2.4-3.9*	*2.1-3.6*		*2.4-4.1*	*2.2-3.8*
	Upper		38-57	36-57		34-56	32-59		41-70	40-76

(G) **Corticosterone** (pmol/L) reference intervals, based on samples ≥40 µL

Groups	CI	All samples			Median of biological replicates			Highest value of biological replicates		
		**N**	**Parametric**	**Robust**	**N**	**Parametric**	**Robust**	**N**	**Parametric**	**Robust**
All infants		124	*14*-1144	*9.8*-920	72	*19*-915	*13*-763	72	*17*-1713	*10*-1433
	Lower		*11-19*	*7-13*		*13-26*	*8.6-18*		*11-25*	*6.8-14*
	Upper		862-1519	620-1274		659-1272	486-1134		1158-2533	748-2390
GA: ≥37 w.		40	*11*-889	*7.6*-812	29	*12*-1101	*6.5*-1052	29	*13*-1246	*9.8*-1342
	Lower		*6.9-19*	*4.3-11*		*6.4-21*	*3.1-10*		*7-24*	*6-19*
	Upper		542-1457	419-1533		601-2017	474-2399		677-2292	647-3118

Groups	CI	All samples			Median of biological replicates			Highest value of biological replicates		
		N	Parametric	Robust	N	Parametric	Robust	N	Parametric	Robust
GA: 32 to <37 w		59	*16*-1272	*12*-1148	30	*32*-632	*24*-588	30	*22*-1898	*9.3*-1486
	Lower		*11-25*	*8-19*		*22-47*	*14-38*		*12-40*	*4.1-13*
	Upper		847-1909	662-1962		428-933	347-1039		1059-3401	539-2929
GA: 28 to <32 w.		14			9			9		
GA: <28 w.		11			4			4		

(H) **Aldosterone** (pmol/L) reference intervals, based on samples ≥1 µL

Groups	CI	All samples			Median of biological replicates			Highest value of biological replicates		
		N	Parametric	Robust	N	Parametric	Robust	N	Parametric	Robust
All infants		362	197.3-3979.8	206.8-4365.6	164	210.2-3828.2	211.6-4201.6	164	240.7-4739.2	249.2-5255.6
	Lower		176.2-220.9	180.8-236.4		178.7-247.3	174.6-249.2		203.7-284.5	200.3-303.4
	Upper		3553.7-4456.9	3930.7-4881.5		3254-4503.7	3664.1-4928.3		4010.8-5599.8	4468.8-6120.3

(I) **DHEA** (nmol/L) reference intervals, based on samples ≥40 µL

**Groups**	**CI**	**All samples**			**Median of biological replicates**			**Highest value of biological replicates**		
		**N**	**Parametric**	**Robust**	**N**	**Parametric**	**Robust**	**N**	**Parametric**	**Robust**
All infants		121	*0.1*-2.7	*0.1*-2.8	72	*0.11*-2.5	*0.09*-2.4	72	*0.12*-3.2	*0.11*-3.4
	Lower		*0.08-0.13*	*0.08-0.12*		*0.08-0.14*	*0.07-0.12*		*0.09-0.15*	*0.09-0.15*
	Upper		2.2-3.3	2.2-3.7		1.9-3.2	1.7-3.2		2.4-4.3	2.5-4.9
GA: ≥37 w.		40	*0.07*-1.8	*0.07*-2	29	*0.08*-2	*0.07*-2.2	29	*0.08*-2.2	*0.08*-2.5
	Lower		*0.05-0.11*	*0.05-0.1*		*0.05-0.12*	*0.04-0.11*		*0.05-0.13*	*0.04-0.12*
	Upper		1.3-2.6	1.3-3.1		1.3-3.1	1.3-3.8		1.4-3.4	1.5-4.4
GA: 32 to <37 w		59	*0.13*-2.1	*0.13*-2.2	31	*0.15*-1.7	*0.13*-1.8	31	*0.16*-2.6	*0.14*-2.8
	Lower		*0.1-0.17*	*0.09-0.17*		*0.11-0.21*	*0.09-0.19*		*0.11-0.23*	*0.1-0.2*
	Upper		1.6-2.7	1.7-3		1.3-2.4	1.3-2.5		1.8-3.7	2-4.1
GA: 28 to <32 w.		14			9			9		
GA: <28 w.		8			3			3		

(J) **DHEAS** (µmol/L) reference intervals, based on samples ≥30 µL

**Groups**	**CI**	**All samples**			**Median of biological replicates**			**Highest value of biological replicates**		
		**N**	**Parametric**	**Robust**	**N**	**Parametric**	**Robust**	**N**	**Parametric**	**Robust**
All infants		145	*0.0029*–0.1046	*0.0027*–0.1038	80	*0.003–0.1032*	*0.0027*–0.1024	80	*0.0032*–0.1201	*0.0029*–0.1359
	Lower		*0.0023–0.0038*	*0.0022–0.0034*		*0.0022–0.004*	*0.002–0.0035*		*0.0023–0.0042*	*0.0022–0.0039*
	Upper		*0.0845*–0.1295	*0.0818*–0.1338		*0.0777*–0.1371	*0.0724*–0.1469		*0.0858*–0.1738	*0.0884*–0.2003
Hours since birth: ≤48		33	*0.005*–0.1587	*0.0043*–0.1711	22	*0.0061*–0.1533	*0.0047*–0.1755	22	*0.0067*–0.1654	*0.0055*–0.1989
	Lower		*0.0033–0.0077*	*0.0028–0.0083*		*0.0037–0.01*	*0.0029–0.0073*		*0.0041–0.011*	*0.0034–0.0093*
	Upper		0.1031–0.2442	0.1107–0.2908		*0.0937*–0.2509	0.102–0.382		0.1014–0.2899	0.1148–0.3588
Hours since birth: >48		112	*0.0027–0.0846*	*0.0025–0.0838*	63	*0.0026–0.0896*	*0.0024–0.0929*	63	*0.0027*–0.1144	*0.0025*–0.1228
	Lower		*0.0021–0.0034*	*0.002–0.0032*		*0.0019–0.0036*	*0.0017–0.0032*		*0.0018–0.0037*	*0.0018–0.0034*
	Upper		*0.067*–0.1068	*0.0839*–0.1096		*0.065*–0.1235	*0.0839*–0.1392		*0.0814*–0.1607	*0.087*–0.1885
Girls		80	*0.003–0.086*	*0.003–0.0921*	41	*0.003–0.0741*	*0.003–0.0843*	41	*0.0028*–0.1126	*0.0028*–0.1289

Groups	CI	All samples			Median of biological replicates			Highest value of biological replicates		
		N	Parametric	Robust	N	Parametric	Robust	N	Parametric	Robust
	Lower		*0.0023–0.0039*	*0.0022–0.0039*		*0.0021–0.0043*	*0.0019–0.0045*		*0.0018–0.0044*	*0.0018–0.0044*
	Upper		*0.0657*–0.1128	*0.0702*–0.123		*0.0518*–0.108	*0.0554*–0.1325		*0.0747*–0.1607	*0.0856*–0.2099
Boys		65	*0.0028*–0.1313	*0.0024*–0.1267	39	*0.0031*–0.1391	*0.0024*–0.1417	39	*0.0035*–0.1485	*0.0028*–0.1527
	Lower		*0.002–0.004*	*0.0019–0.0033*		*0.002–0.0048*	*0.0017–0.0038*		*0.0023–0.0053*	*0.002–0.0041*
	Upper		*0.0833*–0.1846	*0.0822*–0.2015		*0.0899*–0.2152	*0.0834*–0.281		*0.0865*–0.2285	*0.0893*–0.281

(K) **Androstenedione** (pmol/L) reference intervals, based on samples ≥20 µL

Groups	CI	All samples			Median of biological replicates			Highest value of biological replicates		
		N	Parametric	Robust	N	Parametric	Robust	N	Parametric	Robust
All infants		163	*39.5*-1752.3	*36.2*-1698.1	82	*38.8*-1595.9	*34*-1514.5	82	*39.9*-2111.6	*36*-2082
	Lower		*31.9-48.9*	*28.6-46.2*		*28.9-52.1*	*24.7-46.1*		*29.1-54.6*	*26.5-48.3*
	Upper		1416.2-2168.2	1311.3-2237.5		1189-2141.9	1079.4-2095.8		1542-2891.5	1491.6-2923.4
GA: ≥37 w.		45	*38.4*-727.6	*33.6*-753.5	34	*34.4*-851.7	*30.1*-917.5	34	*34.9*-930	*31.4*-1014.2

Groups	CI	All samples			Median of biological replicates			Highest value of biological replicates		
		N	Parametric	Robust	N	Parametric	Robust	N	Parametric	Robust
	Lower		*28-52.5*	*24.2-44.8*		*23.2-51*	*20.2-42.5*		*23.3-52.3*	*20.9-45.9*
	Upper		531.2-996.6	538.1-1116		573.9-1263.9	617.2-1440.8		621.2-1392.3	698.5-1525.9
GA: 32 to <37 w		82	*40.7*-1889	*39*-1955.8	34	*52.8*-1453.6	*47.5*-1566.5	34	*55.3*-2118	*49.9*-2334.2
	Lower		*30.1-55.2*	*26.4-56.3*		*35.1-79.4*	*31.8-70.1*		*35.3-86.5*	*31.6-77*
	Upper		1394.1-2559.7	1266.4-2997.9		967-2185.2	1038-2545.5		1352.6-3316.4	1506.3-3847.6
GA: 28 to <32 w.		22	*80.9*-666.4	*60.9*-654.8	9			9		
	Lower		*58.6-111.7*	*38.2-83.6*						
	Upper		482.7-919.9	420.8-1111						
GA: <28 w.		14			5			5		
Max respiratory support:										
• none		102	*41.5*-1022.3	*38.4*-1006.6	61	*39.8*-968.9	*37.3*-1004.4	61	*38.8*-1214.2	*36.2*-1259.4
	Lower		*33.1-52.1*	*30.5-48.1*		*29.7-53.3*	*27.6-49.2*		*28.3-53.2*	*26-48.3*
	Upper		814.2-1283.4	792.1-1311		722.7-1298.9	745.6-1365.8		885-1665.9	912.2-1759.2
• nasal cannula		20	*85.8*-1115.2	*70.3*-1165	8			8		

Groups	CI	All samples			Median of biological replicates			Highest value of biological replicates		
		N	Parametric	Robust	N	Parametric	Robust	N	Parametric	Robust
	Lower		*56.9-129.4*	*41.7-100.4*						
	Upper		739.1-1682.5	720.8-1854.1						

(L) **Testosterone** (pmol/L) reference intervals, based on samples ≥20µL

Groups	CI	All samples			Median of biological replicates			Highest value of biological replicates		
		N	Parametric	Robust	N	Parametric	Robust	N	Parametric	Robust
All infants		157	*3.3*-262.5	*2.8*-243.8	82	*4.2*-215.3	*3.4*-200.9	82	*4.4*-278.8	*3.6*-251.6
	Lower		*2.5-4.2*	*2.2-3.7*		*3.1-5.7*	*2.3-4.7*		*3.2-6.2*	*2.5-5.3*
	Upper		204.2-337.4	183.1-327.1		157.6-294	135.4-299.7		200.9-386.9	167.2-380.5
Hours since birth: ≤48		35	*2.9*-577.2	*2*-569.6	23	*3.4*-629.2	*2.1*-714.6	23	*4.1*-660.4	*2.3*-683.8
	Lower		*1.5-5.5*	*1.2-4.2*		*1.5-7.4*	*1-4.4*		*1.9-8.8*	*0.9-3.9*
	Upper		303.8-1096.6	250-1415.1		287.8-1375.3	286.5-2318		309.4-1409.7	261.9-1937.7
Hours since birth: >48		122	*3.5*-200.2	*3.3*-199.9	63	*4.6*-155.8	*3.9*-152.7	63	*4.8*-199.3	*4*-189.2
	Lower		*2.7-4.6*	*2.5-4.3*		*3.4-6.3*	*2.8-5.7*		*3.4-6.7*	*2.7-5.8*
	Upper		154-260.2	151.5-267.2		113.3-214.1	104.4-232.2		142.4-279	126.9-285.9
Girls		81	*2.9*-124.1	*2.5*-122.9	41	*3.9*-99.6	*3.3*-104.2	41	*4*-133.3	*3.1*-138.1

Groups	CI	All samples			Median of biological replicates			Highest value of biological replicates		
		**N**	**Parametric**	**Robust**	**N**	**Parametric**	**Robust**	**N**	**Parametric**	**Robust**
	Lower		*2.1-3.9*	*1.8-3.3*		*2.7-5.7*	*2.3-4.7*		*2.7-5.9*	*1.9-4.8*
	Upper		92-167.5	87.2-179.4		69.4-143	70.3-168.7		90-197.3	89-240.9
Boys		76	*5.4*-399.1	*4.4*-367.8	41	*6.4*-326.1	*4.7*-316.5	41	*6.9*-417.6	*4.9*-373.1
	Lower		*3.8-7.7*	*3.2-6.2*		*4.1-9.9*	*3.1-7.3*		*4.3-10.9*	*3-7.3*
	Upper		280.2-568.3	238.4-569.9		209.9-506.6	181.5-588.2		263.6-661.5	190.9-680

Reference intervals (RI) are calculated with parametric and robust methods based on either all available samples, or median value for each individual, or on the highest value for each individual. We report both parametric and robust RIs for groups with ≥20 values, both computed on log-transformed values and transformed back. Parametric method is the recommended option when ≥120 values is available, robust method is used for smaller sample size, however, following Horn, Pesce & Copeland, 1998, we include parametric results for all subgroups for comparison purposes. For groups with <20 samples, RIs are not computed. For each hormone, we assessed the threshold saliva volume that does not have influence on the hormone concentration and used samples above that volume for RI construction. We used all samples, also those below LLoQ, for RI construction to avoid bias to the right. Therefore, for some metabolites, the limits of RI are below LLoQ (highlighted with *Italics*). Due to a small sample size and lack of studies of hormonal trends in infants, we adopted conservative approach to outlier removal (even though we are aware it is a standard procedure in the reference interval estimation). Not more than two outliers were removed for some hormones. CI, confidence intervals; GA, gestational age; w., weeks.

Supplementary figures: Figure 3 alternative with polynomial trend line

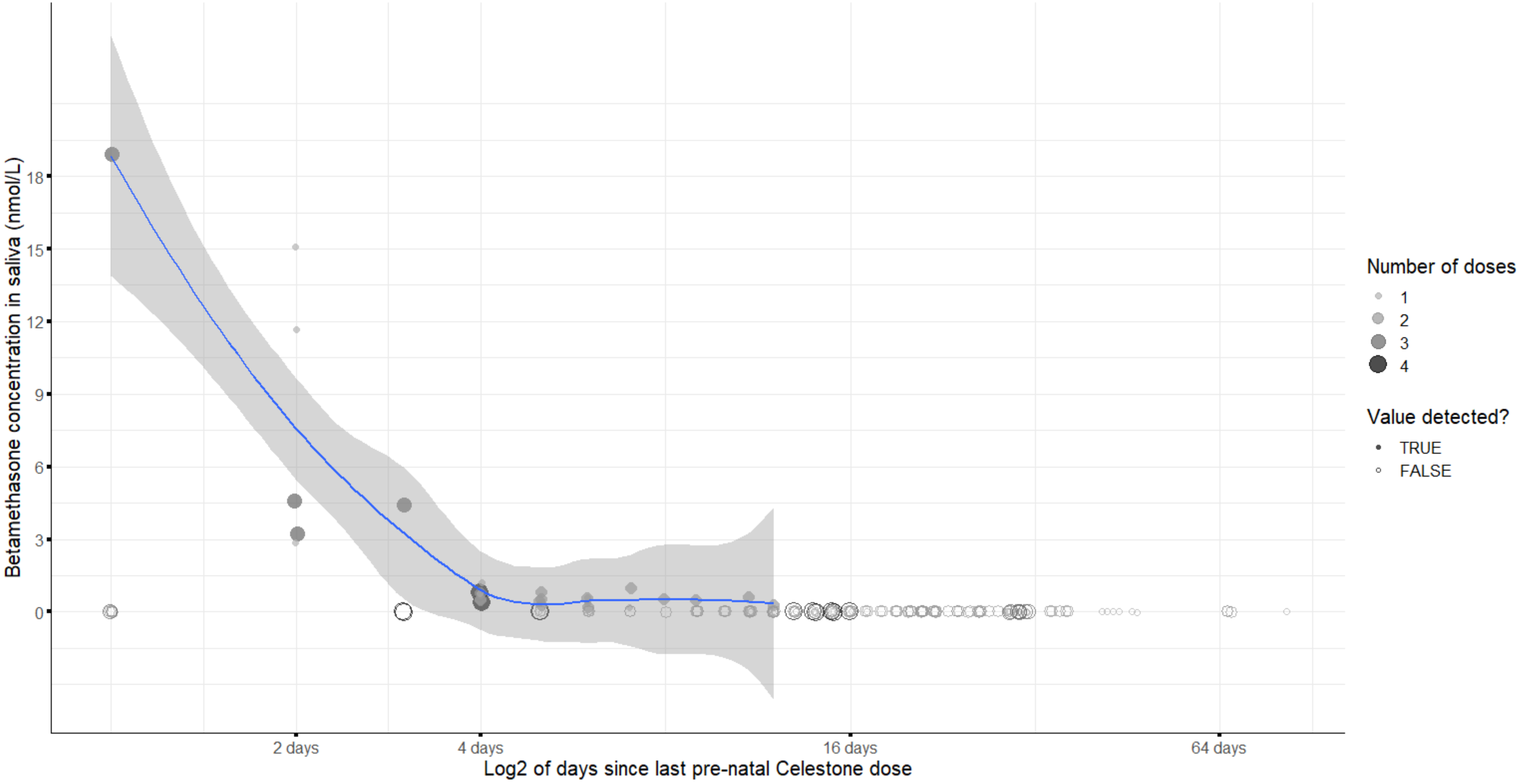

Supplementary figures: boxplots showcasing conditions that have significant effect in adjusted linear mixed models

All the supplementary figures show salivary concentration of adrenal hormones on Y-axis. The concentrations of 17OH-pregnenolone, 17OH-progesterone , 11-deoxycortisol , deoxycorticosterone, corticosterone, aldosterone, androstenedione and testosterone are in pmol/L, of cortisone, cortisol and DHEA in nmol/L and for DHEAS in µmol/L. p-value in all the plots are from the multiply adjusted models (Table 3). Only p-values corresponding to significant effects are reported in the plots. Please note that p-value is not from direct comparison but from an linear mixed model adjusted for all studied effects.

(A) Highest grade of respiratory support

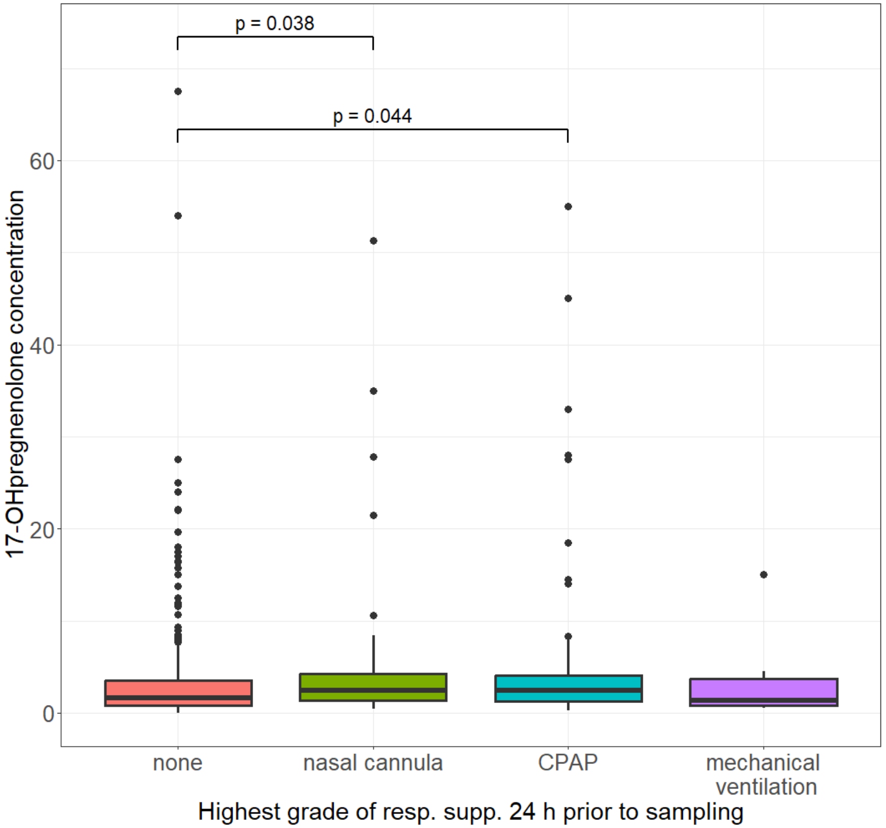

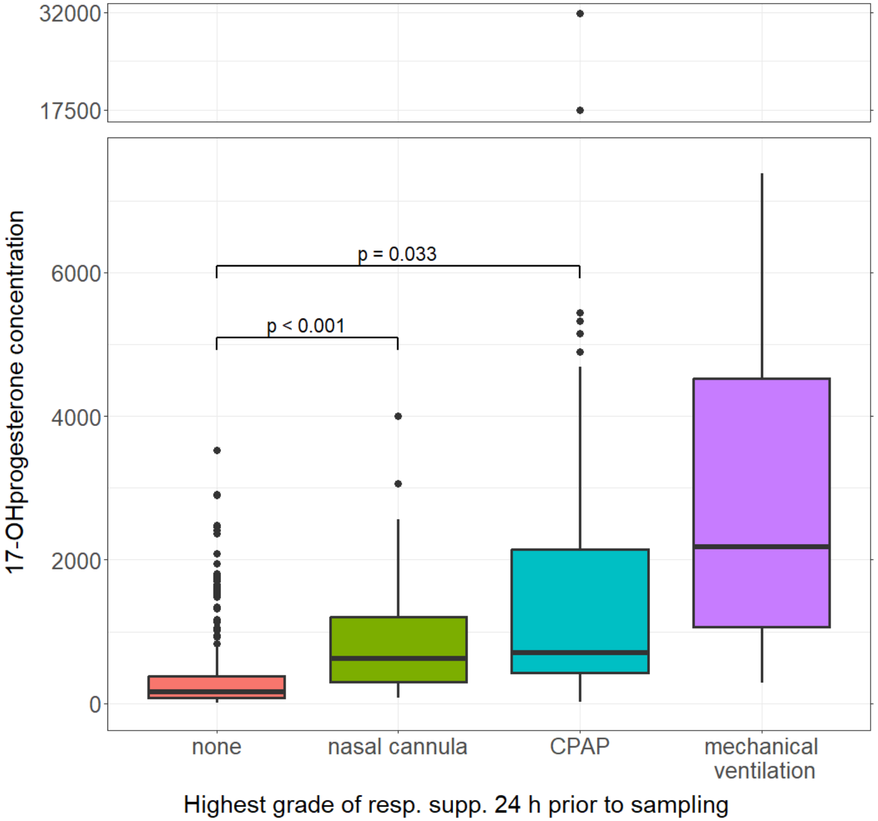

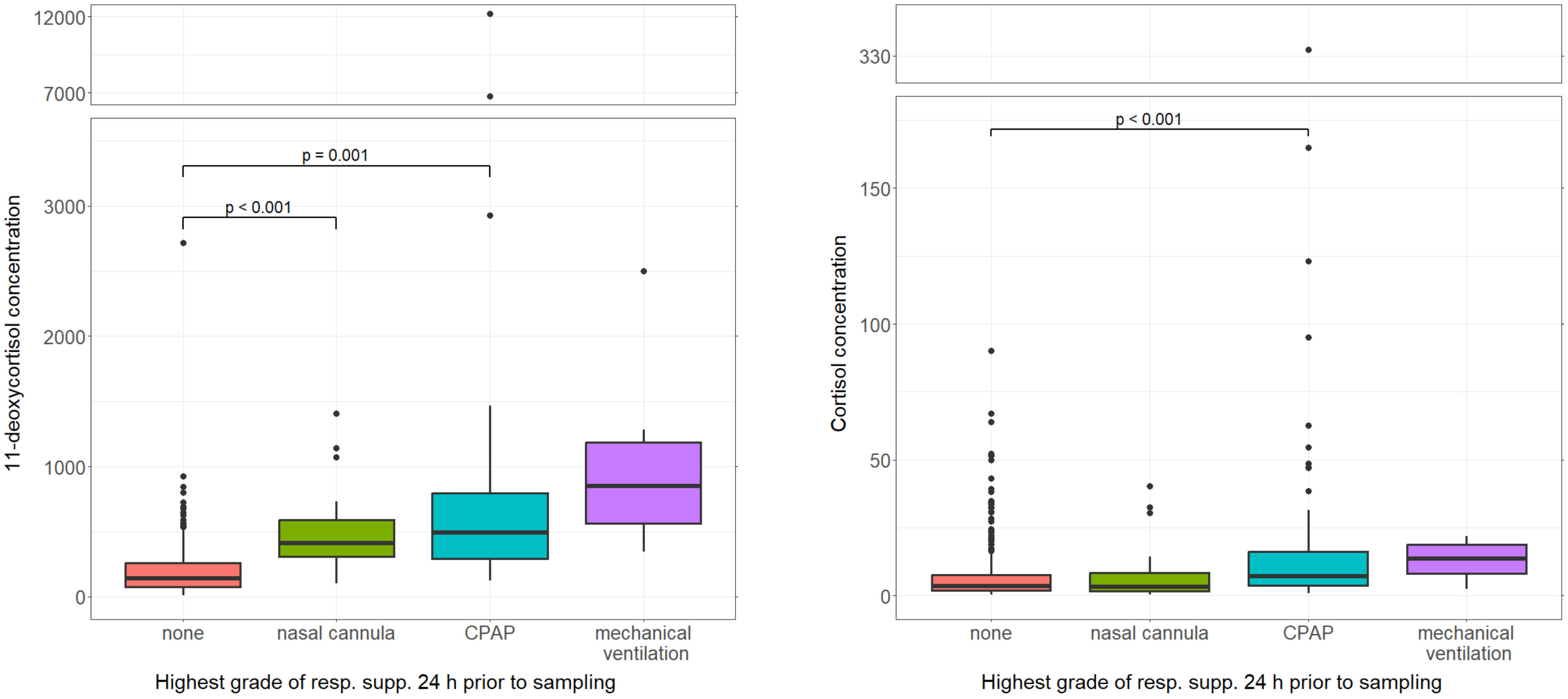

12000
7000
3000
2000
1000
0
p = 0.001
p < 0.001
11-deoxycortisol concentration
none
nasal cannula
CPAP
mechanical ventilation
Highest grade of resp. supp. 24 h prior to sampling
330
150
100
50
0
p < 0.001
Cortisol concentration
none
nasal cannula
CPAP
mechanical ventilation
Highest grade of resp. supp. 24 h prior to sampling

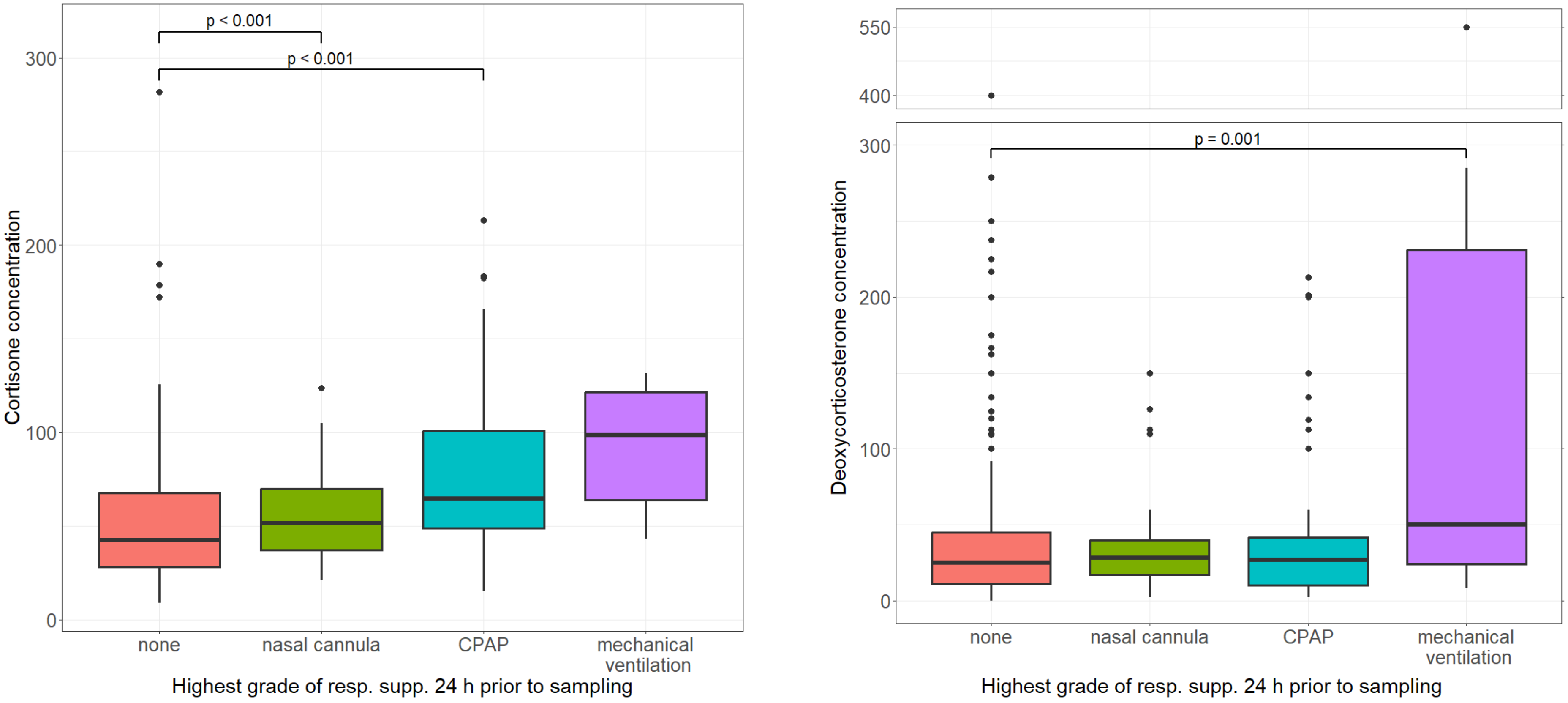
p < 0.001
p < 0.001
Cortisone concentration
300
200
100
0
none
nasal cannula
CPAP
mechanical ventilation
Highest grade of resp. supp. 24 h prior to sampling
550
400
p = 0.001
Deoxycorticosterone concentration
300
200
100
0
none
nasal cannula
CPAP
mechanical ventilation
Highest grade of resp. supp. 24 h prior to sampling

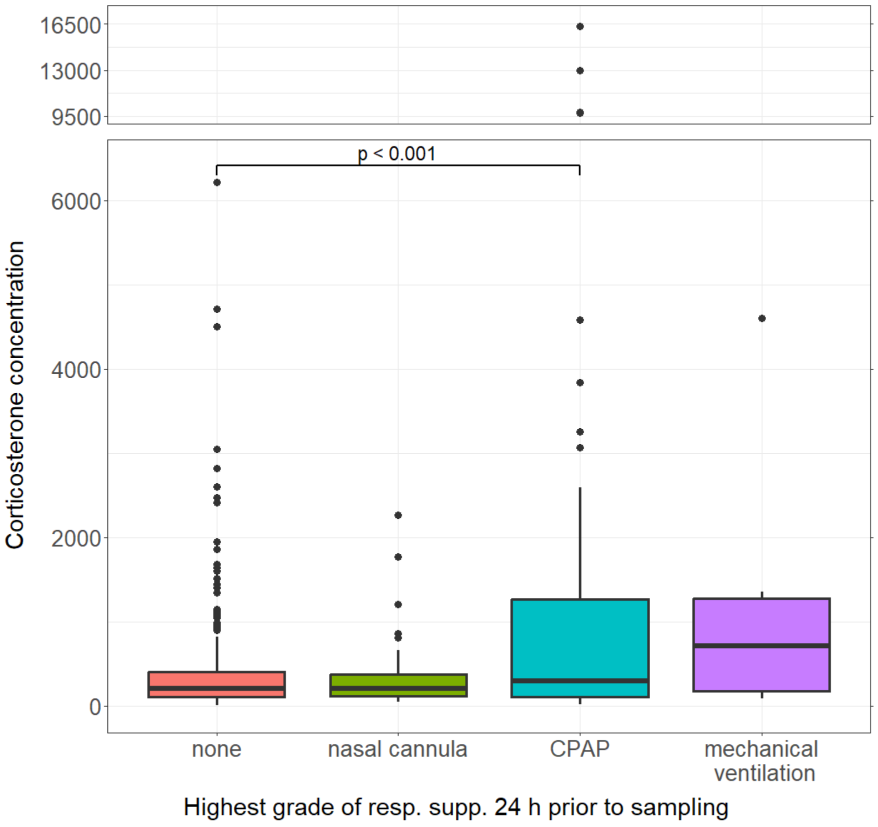

16500
13000
9500
p < 0.001
6000
4000
2000
0
Corticosterone concentration
none
nasal cannula
CPAP
mechanical ventilation
Highest grade of resp. supp. 24 h prior to sampling

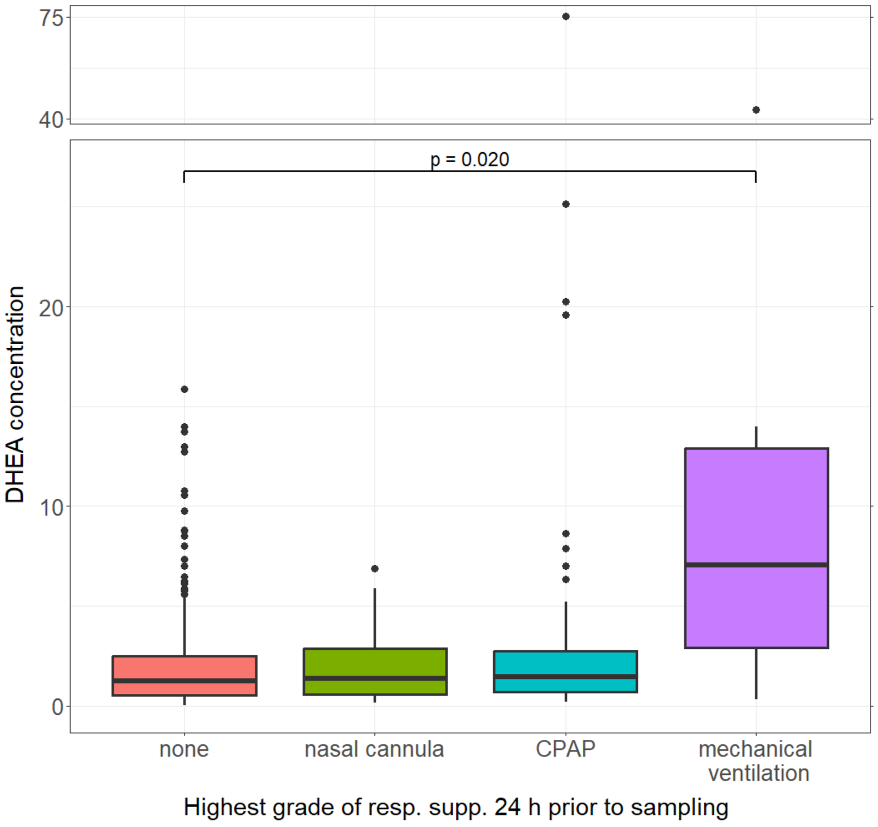

75
40
p = 0.020
20
10
0
DHEA concentration
none
nasal cannula
CPAP
mechanical ventilation
Highest grade of resp. supp. 24 h prior to sampling

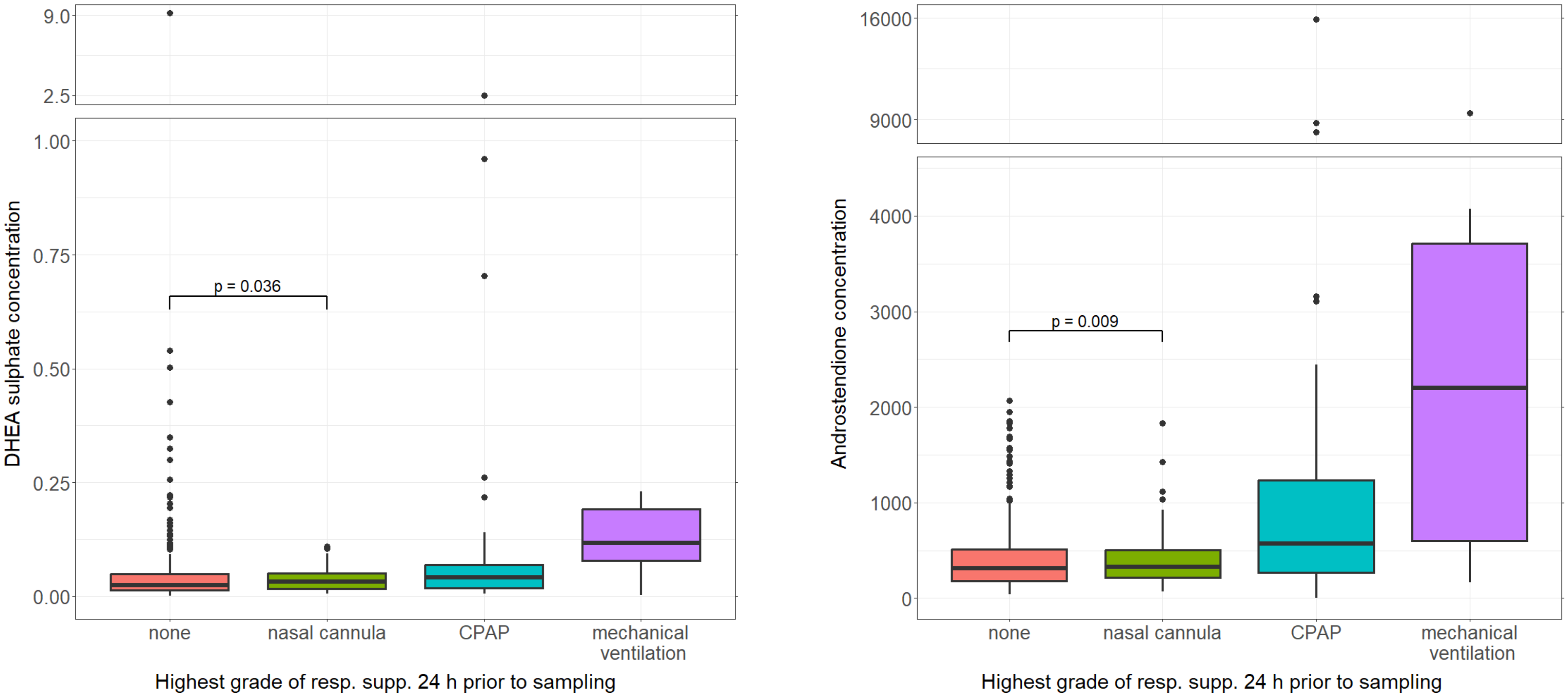
9.0
2.5
1.00
0.75
0.50
0.25
0.00
p = 0.036
DHEA sulphate concentration
none
nasal cannula
CPAP
mechanical ventilation
Highest grade of resp. supp. 24 h prior to sampling
16000
9000
4000
3000
2000
1000
0
p = 0.009
Androstendione concentration
none
nasal cannula
CPAP
mechanical ventilation
Highest grade of resp. supp. 24 h prior to sampling

(B) Hours since birth

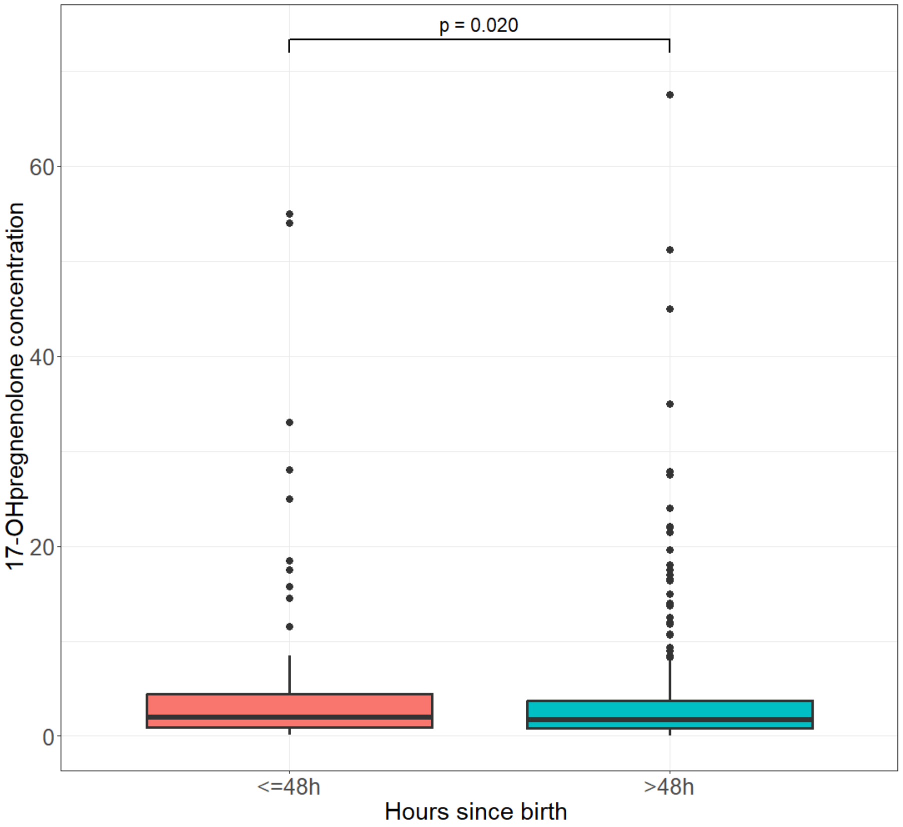

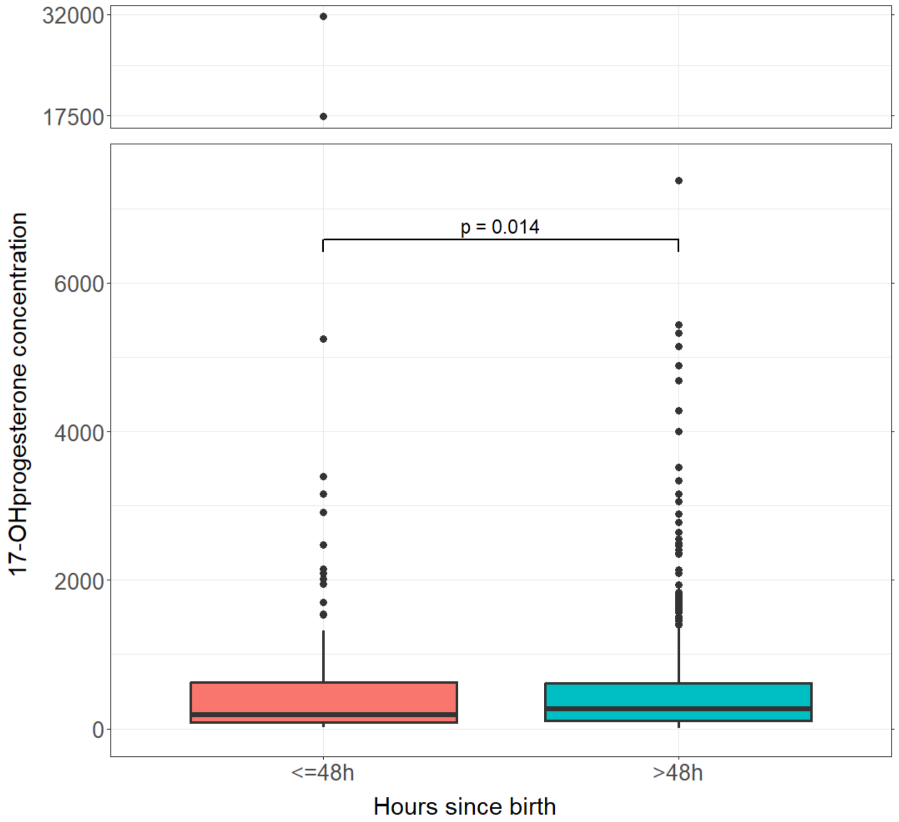

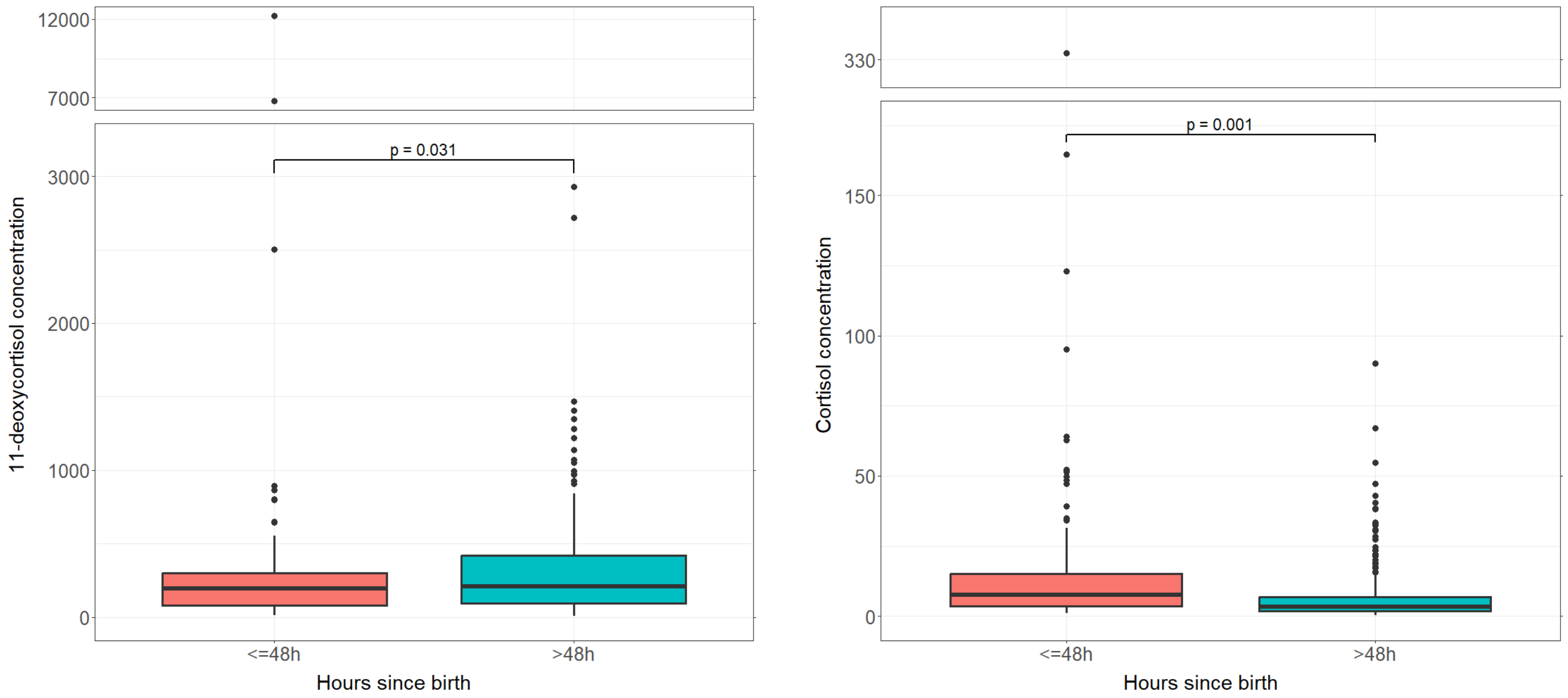

11-deoxycortisol concentration
12000
7000
3000
2000
1000
0
p = 0.031
<=48h
>48h
Hours since birth
Cortisol concentration
330
150
100
50
0
p = 0.001
<=48h
>48h
Hours since birth

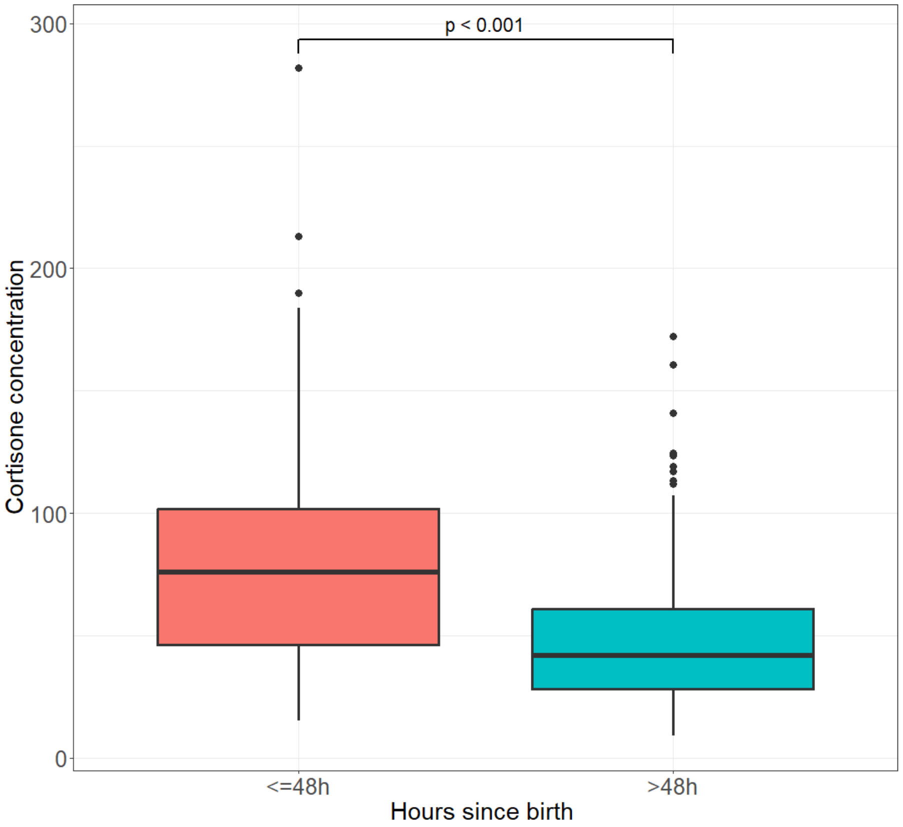
p < 0.001
300
200
100
0
Cortisone concentration
<=48h
>48h
Hours since birth

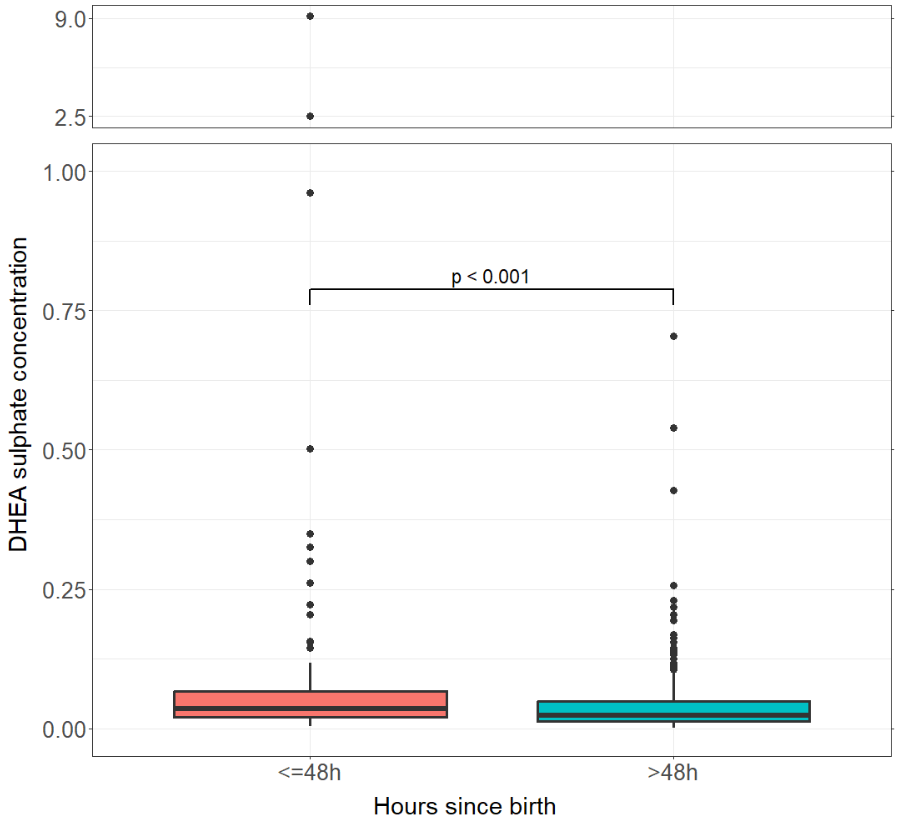
9.0
2.5
1.00
0.75
0.50
0.25
0.00
p < 0.001
DHEA sulphate concentration
<=48h
>48h
Hours since birth

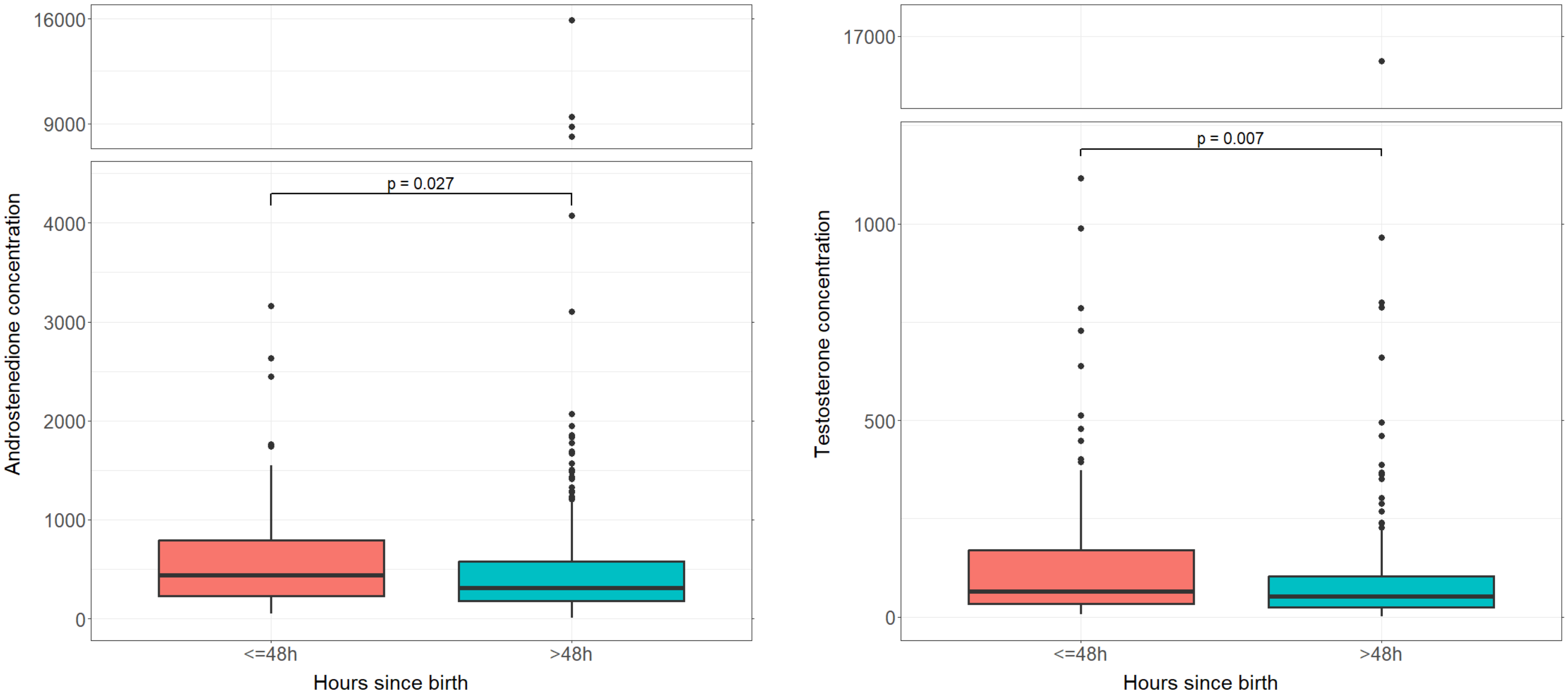

16000
9000
p = 0.027
4000
3000
2000
1000
0
Androstenedione concentration
<=48h
>48h
Hours since birth
17000
p = 0.007
1000
500
0
Testosterone concentration
<=48h
>48h
Hours since birth

(C) Gestational age

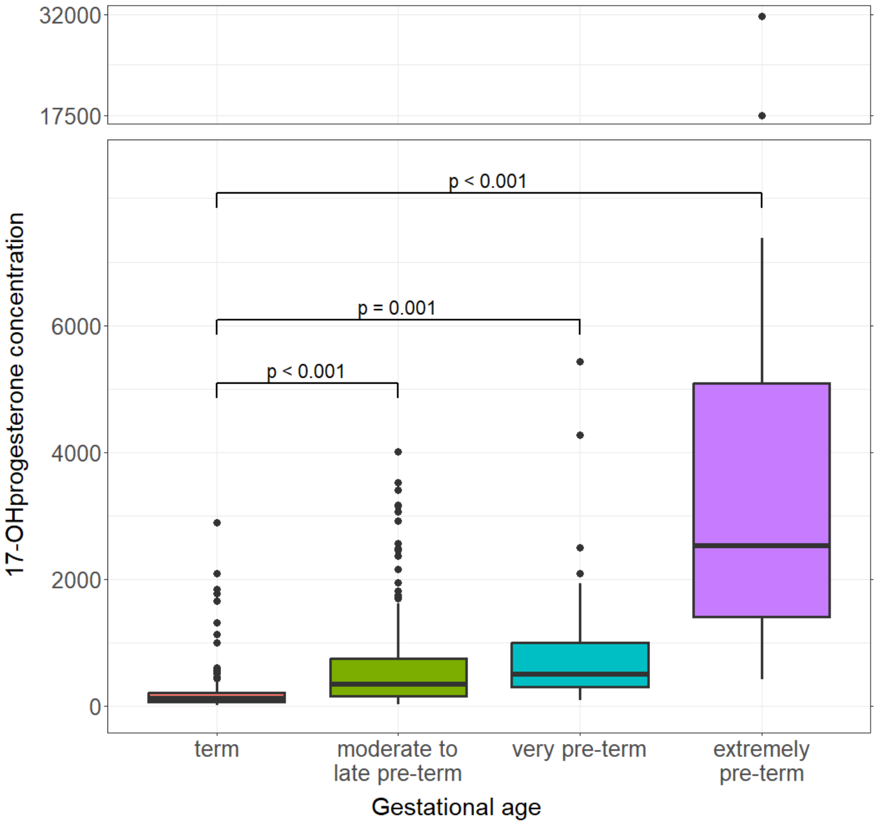

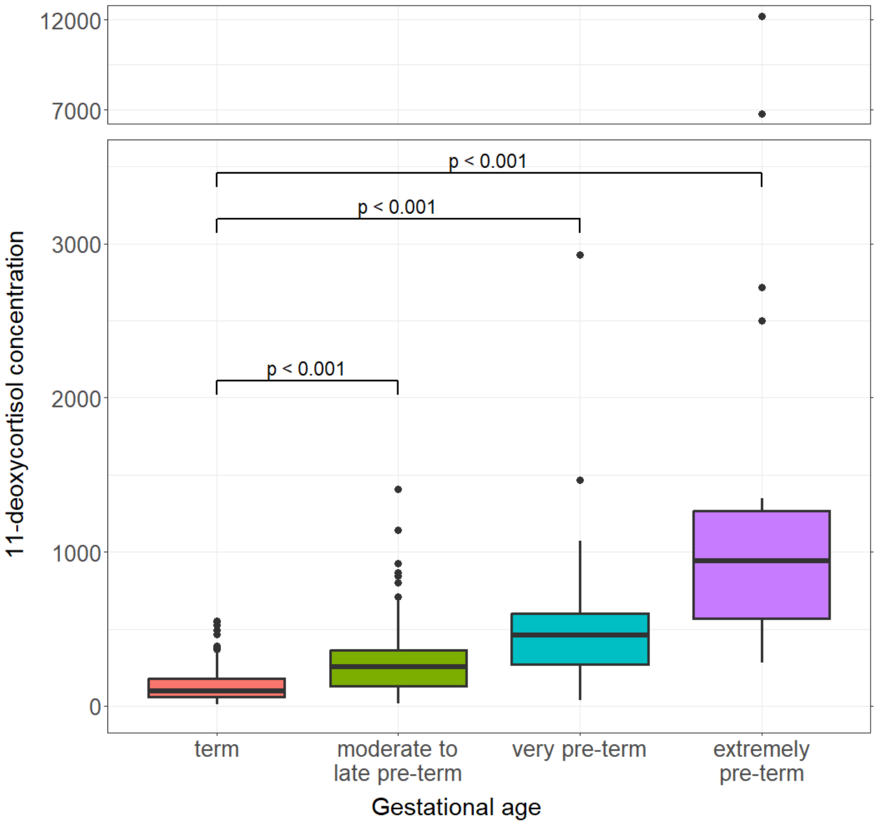

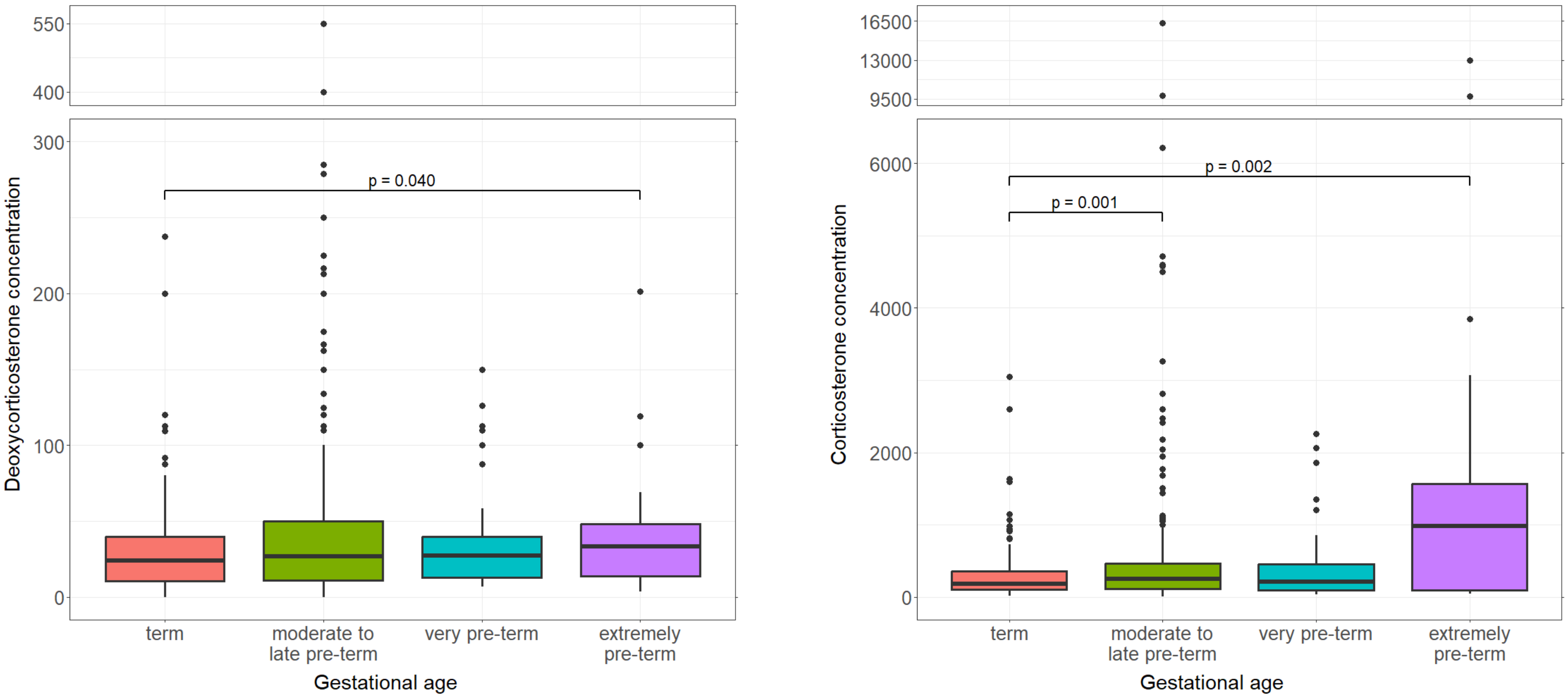
550
400
300
200
100
0
p = 0.040
Deoxycorticosterone concentration
term
moderate to late pre-term
very pre-term
extremely pre-term
Gestational age
16500
13000
9500
6000
4000
2000
0
p = 0.002
p = 0.001
Corticosterone concentration
term
moderate to late pre-term
very pre-term
extremely pre-term
Gestational age

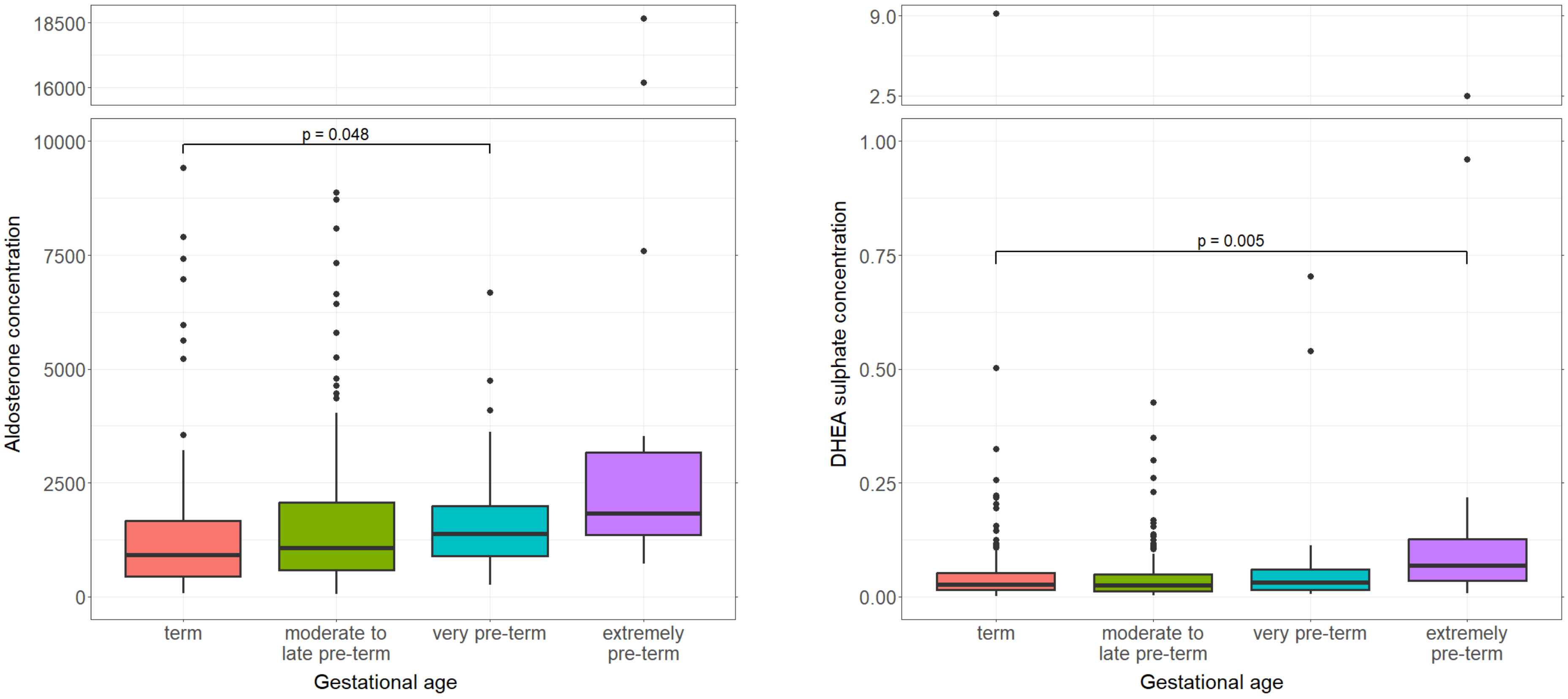
18500
16000
10000
7500
5000
2500
0
p = 0.048
Aldosterone concentration
term
moderate to late pre-term
very pre-term
extremely pre-term
Gestational age
9.0
2.5
1.00
0.75
0.50
0.25
0.00
p = 0.005
DHEA sulphate concentration
term
moderate to late pre-term
very pre-term
extremely pre-term
Gestational age

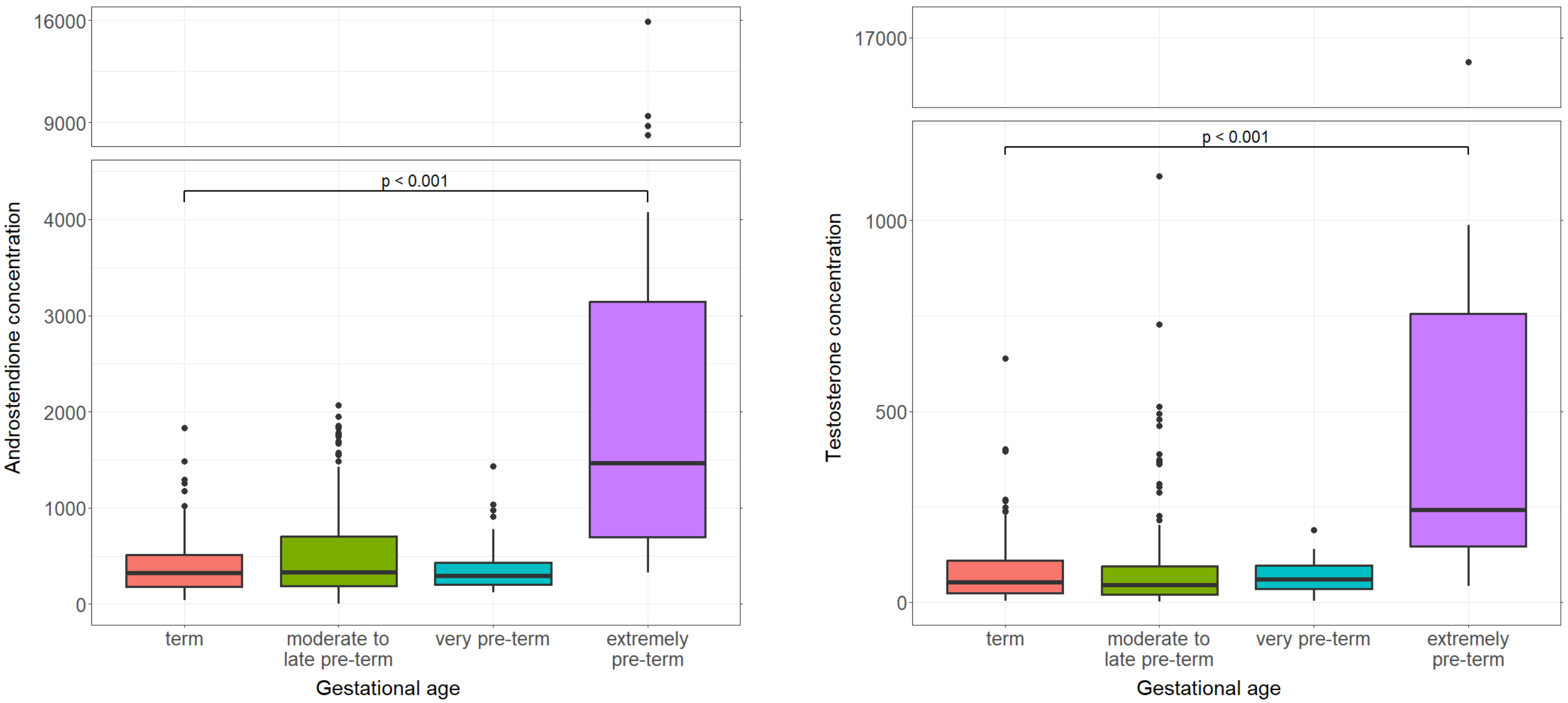
16000
9000
4000
3000
2000
1000
0
p < 0.001
Androstendione concentration
term
moderate to late pre-term
very pre-term
extremely pre-term
Gestational age
17000
1000
500
0
p < 0.001
Testosterone concentration
term
moderate to late pre-term
very pre-term
extremely pre-term
Gestational age

(D) Sex

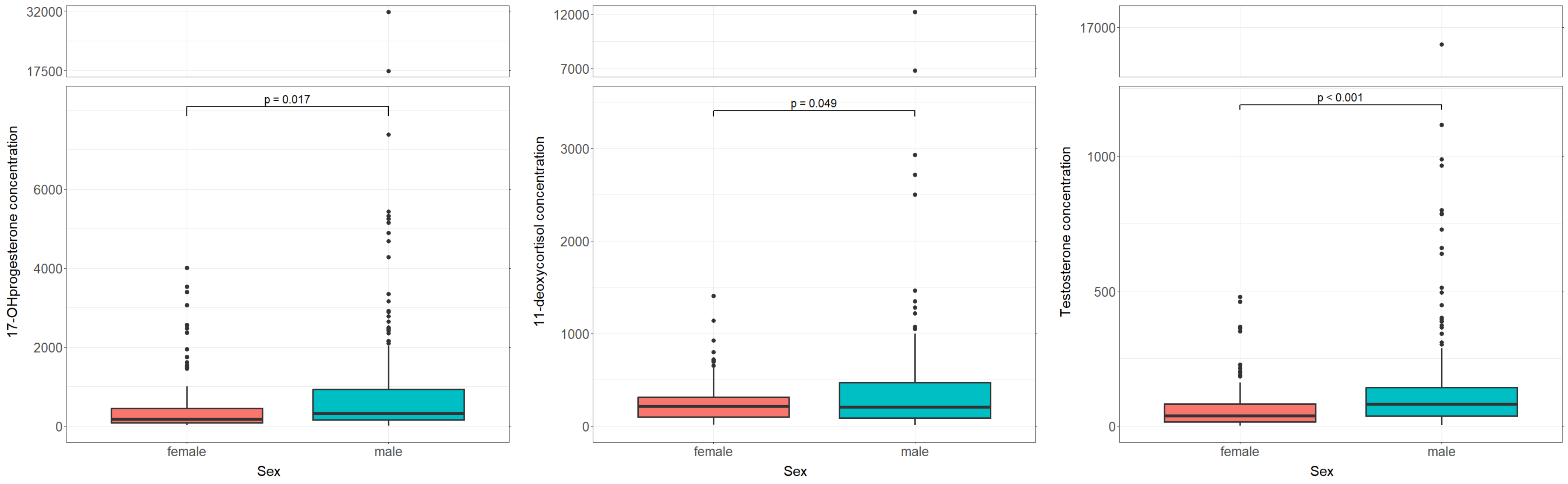

32000
17500
p = 0.017
6000
4000
2000
0
17-OHprogesterone concentration
female
male
Sex
12000
7000
p = 0.049
3000
2000
1000
0
11-deoxycortisol concentration
female
male
Sex
17000
p < 0.001
1000
500
0
Testosterone concentration
female
male
Sex

(E) Gestational age and sex

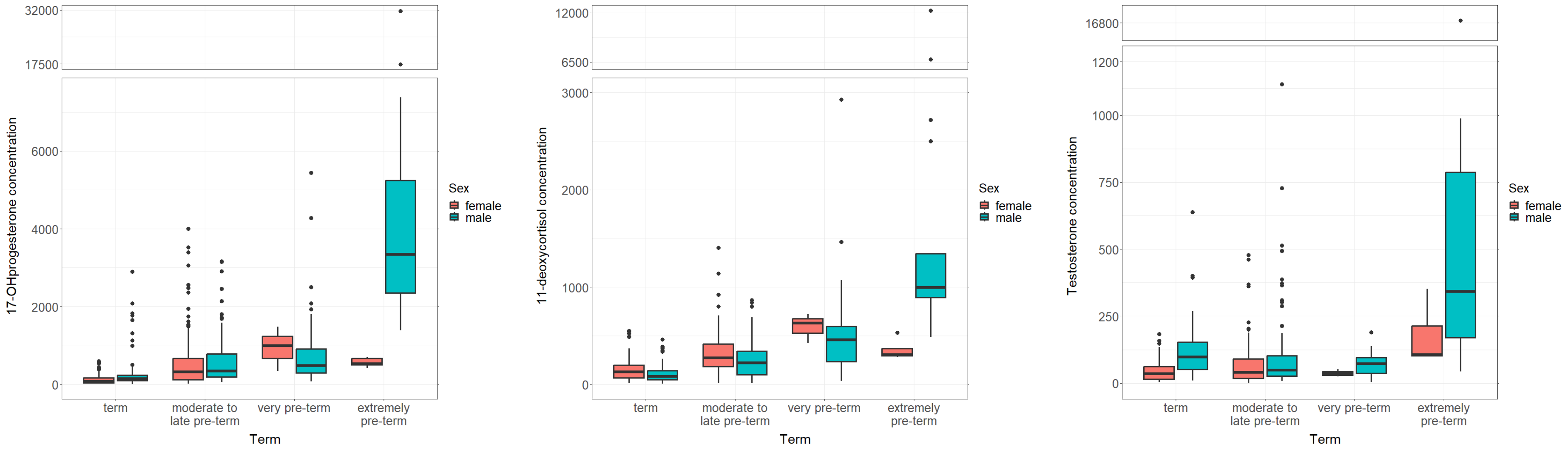

Supplement: Supplementary file 1 [file Datasheet1.pdf]
