# Supplementary material for: Neonatal reference intervals for salivary steroids: focus on prematurity, stressful events and perinatal betamethasone
Source: Front Pediatr. 2026 Jun 16;14:1808446. doi: 10.3389/fped.2026.1808446 (PMC13281276; doi:10.3389/fped.2026.1808446)
Supplement: Supplementary file 2 [file Presentation1.pptx]

## Slide 1
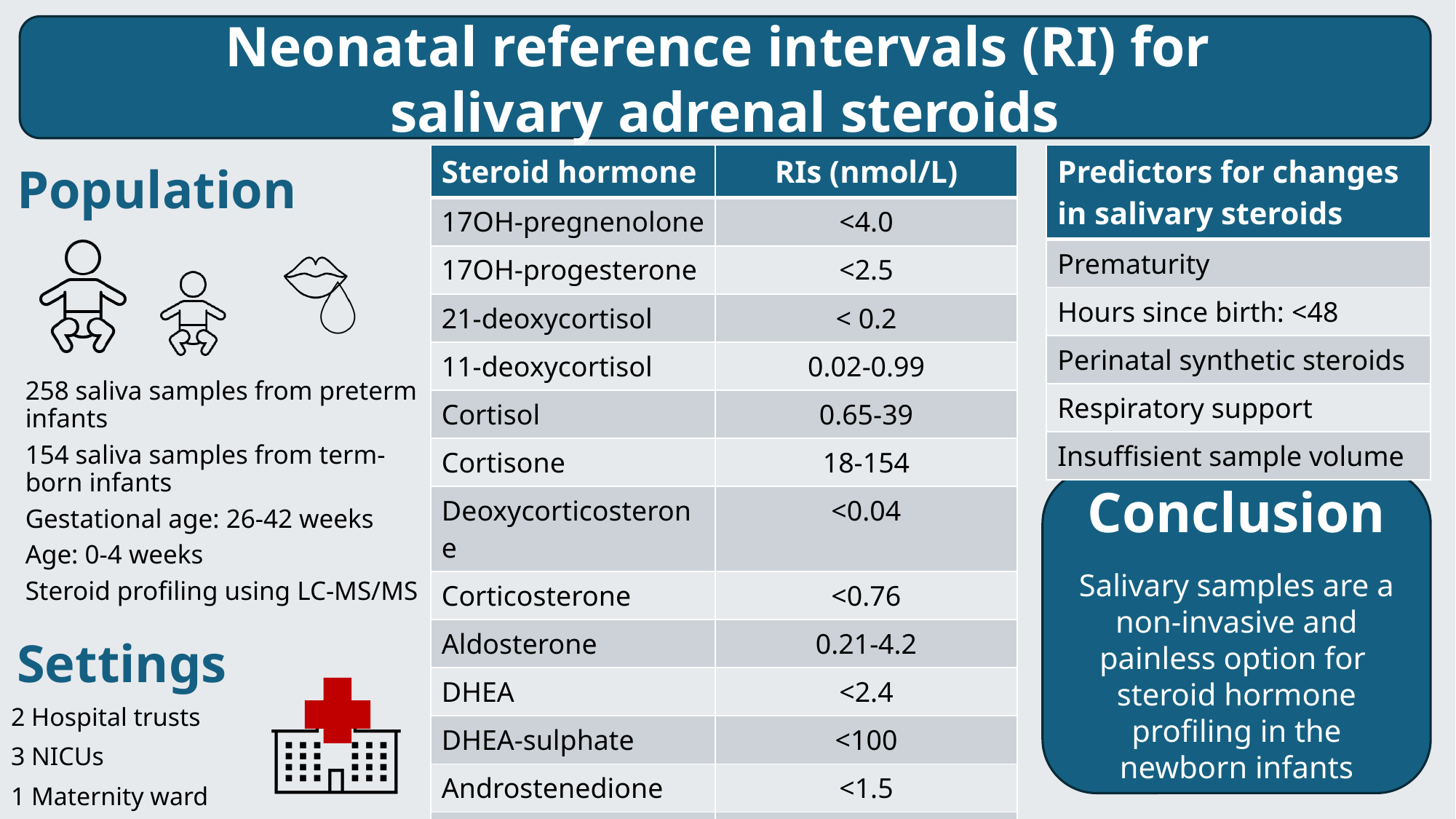

Neonatal reference intervals (RI) for
salivary adrenal steroids
| Steroid hormone | RIs (nmol/L) |
| --- | --- |
| 17OH-pregnenolone | <4.0 |
| 17OH-progesterone | <2.5 |
| 21-deoxycortisol | < 0.2 |
| 11-deoxycortisol | 0.02-0.99 |
| Cortisol | 0.65-39 |
| Cortisone | 18-154 |
| Deoxycorticosterone | <0.04 |
| Corticosterone | <0.76 |
| Aldosterone | 0.21-4.2 |
| DHEA | <2.4 |
| DHEA-sulphate | <100 |
| Androstenedione | <1.5 |
| Testosterone, girls | <0.10 |
| Testosterone, boys | <0.32 |
| Predictors for changes in salivary steroids |
| --- |
| Prematurity |
| Hours since birth: <48 |
| Perinatal synthetic steroids |
| Respiratory support |
| Insuffisient sample volume |
Population
258 saliva samples from preterm infants
154 saliva samples from term-born infants
Gestational age: 26-42 weeks
Age: 0-4 weeks
Steroid profiling using LC-MS/MS
Conclusion
Salivary samples are a non-invasive and painless option for steroid hormone profiling in the newborn infants
Settings
2 Hospital trusts
3 NICUs
1 Maternity ward
